# Supplementary material for: Inter-valley coherent order and isospin fluctuation mediated superconductivity in rhombohedral trilayer graphene
Source: Nat Commun. 2022 Oct 12;13:6013. doi: 10.1038/s41467-022-33561-w (PMC9556532; doi:10.1038/s41467-022-33561-w)
Supplement: Supplementary file 1 — Supplementary Information [file 41467_2022_33561_MOESM1_ESM.pdf]

Supplementary information: Inter-valley coherent order and isospin fluctuation mediated superconductivity in rhombohedral trilayer graphene

Shubhayu Chatterjee,[1] Taige Wang,[1,2] Erez Berg,[3] and Michael P. Zaletel[1,2]

[1] *Department of Physics, University of California, Berkeley, CA 94720, USA*
[2] *Materials Sciences Division, Lawrence Berkeley National Laboratory, Berkeley, California 94720*
[3] *Department of Condensed Matter Physics, Weizmann Institute of Science, Rehovot 76100, Israel*

Supplementary Note 1. MODEL AND SYMMETRIES

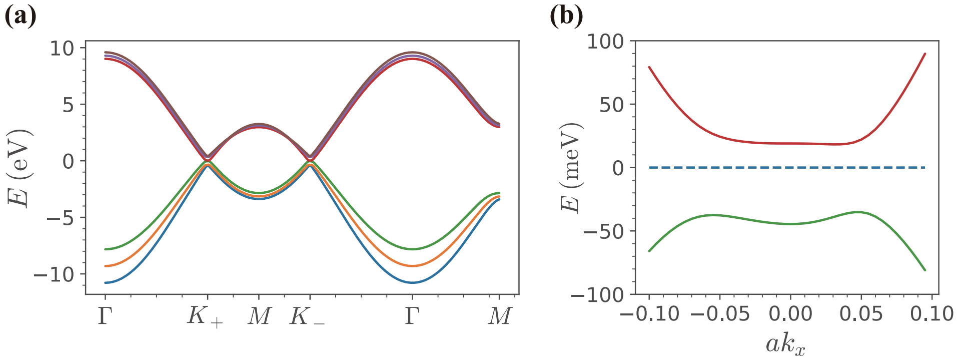

Supplementary Figure 1. (a) Band structure along a high-symmetry line in the BZ for the 6-band model, and (b) Zoom-in near the K point showing the flat spectrum and gapping out of cubic band touching at $\mathbf{K}$, due to the perpendicular electric field quantified by $u = 30\,\text{meV}$.

ABC trilayer graphene or RTG consists of three graphene monolayers, each displaced relative to the next by the same translation vector. Each unit cell consists of six sites (two sublattice sites A_i, B_i of the honeycomb lattice in each layer i), which we index by $(A_1, B_3, B_1, A_2, B_2, A_3)$. Following Ref. 1 and 2, we consider the following 6-band Hamiltonian (per valley, per spin) at low energy to describe the single-particle band structure.

$$H_{6-band} = \begin{pmatrix} \Delta_1+\Delta_2+\delta & \frac{\gamma_2}{2} & v_0\Pi^\dagger & v_4\Pi^\dagger & v_3\Pi & 0 \\ \frac{\gamma_2}{2} & \Delta_2-\Delta_1+\delta & 0 & v_3\Pi^\dagger & v_4\Pi^\dagger & v_0\Pi \\ v_0\Pi & 0 & \Delta_1+\Delta_2 & \gamma_1 & v_4\Pi^\dagger & 0 \\ v_4\Pi & v_3\Pi & \gamma_1 & -2\Delta_2 & v_0\Pi^\dagger & v_4\Pi^\dagger \\ v_3\Pi^\dagger & v_4\Pi^\dagger & v_4\Pi & v_0\Pi & -2\Delta_2 & \gamma_1 \\ 0 & v_0\Pi^\dagger & 0 & v_4\Pi & \gamma_1 & \Delta_2-\Delta_1 \end{pmatrix} \quad (1)$$

where $\Pi = \tau k_x + i k_y$ ($\tau = \pm$ labels valleys), γ_i are the bare hopping matrix elements, $v_i = \sqrt{3} a \gamma_i / 2$ with $a = 0.246$ nm being the graphene lattice constant, δ is an on-site potential only present at the non-dimerized sites A_1 and B_3, and Δ_i account for the potential difference between the layers due to the perpendicular displacement field. The values of all tight binding parameters of our study are chosen from Ref. 2.

The band structure for the 6-band model is shown in Supplementary Figure 1. To analytically understand the symmetries of the problem, it is convenient to project the Hamiltonian onto the two *active* low-energy bands per valley per spin, assuming a small enough density of doped holes or electrons near charge neutrality so that these bands are separated from the remote band by a gap of order γ_1. In other words, most of the spectral weight of the active bands lie on the non-dimerized A_1/B_3 sites, which constitute an effecive sublattice space σ, and the remote bands have spectral weight concentrated on the dimerized sites B_1/A_2 and B_2/A_3. In the σ pseudospin basis, the Hamiltonian can be approximately written as (N = total number of unit cells):

$$H = \sum_{\tau,s,\mathbf{k}} c^\dagger_{\tau,s,\mathbf{k},\sigma} \left([h_\tau(\mathbf{k})]_{\sigma,\sigma'} - \mu\,\delta_{\sigma\sigma'}\right) c_{\tau,s,\mathbf{k},\sigma'} + H_{\text{C}}, \text{ with } [h_\tau(\mathbf{k})]_{\sigma,\sigma'} = \begin{pmatrix} -u & \frac{v^3}{\gamma_1^2}(\tau_z k_x + i k_y)^3 + \frac{\gamma_2}{2} \\ \frac{v^3}{\gamma_1^2}(\tau_z k_x - i k_y)^3 + \frac{\gamma_2}{2} & u \end{pmatrix}_{\sigma\sigma'},$$

$$H_C = \frac{1}{2A}\sum_{\mathbf{q}} V_C(\mathbf{q}) : \rho(\mathbf{q})\rho(-\mathbf{q}) : \text{ and } \rho(\mathbf{q}) = \sum_{\tau,s,\mathbf{k}} c^\dagger_{\tau,s,\mathbf{k}} c_{\tau,s,\mathbf{k}+\mathbf{q}} \quad (2)$$

In Eq. (2), $c^\dagger_{\tau,s,\mathbf{k},\sigma}$ denotes the electron creation operator at momenta $\mathbf{k}$ for valley/spin/sublattice indices $\tau/s/\sigma$ respectively. $u \sim \Delta_1$ is the difference in electrostatic potential in the top and bottom layers due to the perpendicular electric field. $\rho(\mathbf{q})$ is the slowly-varying component of the electron density operator, involving only 'intra-valley' terms. Inter-valley scattering terms, which modulate on the lattice scale, and are suppressed at low densities near charge neutrality for long-range Coulomb interactions, will be discussed later.

For analytical arguments, we will often restrict our attention to the relevant 'active' band (for a given valley and spin index) which is crossed by the chemical potential. Hence, it would be convenient to move to the band-basis, and recast the Hamiltonian in terms of the Bloch-eigenstates of the single-particle Hamiltonian, denoted by $u_{n,\tau,\mathbf{k},s}(\sigma)$. To do so, we write:

$$c^\dagger_{\tau,s,\mathbf{k},\sigma} = \sum_n u^*_{n,\tau,\mathbf{k},s}(\sigma)\psi^\dagger_{n,\tau,s,\mathbf{k}}, \tag{3}$$

where n is the band-index and $\psi^\dagger_{n,\tau,s,\mathbf{k}}$ is the corresponding electron creation operator. In this basis, the free Hamiltonian takes the simple form $\sum_{\mathbf{k},n,s,\tau} \varepsilon_{n,\tau}(\mathbf{k})\psi^\dagger_{n,\tau,s,\mathbf{k}}\psi_{n,\tau,s,\mathbf{k}}$, where $\varepsilon_{n,\tau}(\mathbf{k})$ is the dispersion of the nth band in valley $\tau = \pm$, obtained by diagonalizing the matrix $h_\tau(\mathbf{k})$. To write the interaction term in the band-basis, it is convenient to define form-factors $[\lambda^{\tau\tau'}_{\mathbf{q}}(\mathbf{k})]^{n,n'} = \langle u_{n,\mathbf{k},\tau,s}|u_{n',\tau',\mathbf{k}+\mathbf{q},s}\rangle$, which characterize the overlap of Bloch-wavefunctions. Since we are interested in the slowly-varying part of electron density, $\rho(\mathbf{q})$ involves only 'intra-valley' form factors and takes the following form:

$$\rho(\mathbf{q}) = \sum_{\tau,s,\mathbf{k}} [\lambda^{\tau\tau}_{\mathbf{q}}(\mathbf{k})]^{n,n'}\psi^\dagger_{n,\tau,s,\mathbf{k}}\psi_{n',\tau,s,\mathbf{k}+\mathbf{q}} \tag{4}$$

We note that in this work, we are mainly concerned with physics in the valence band in each valley, corresponding to small hole-doping near charge neutrality. So unless otherwise mentioned in our analytical studies we will fix $n = n' =$ 'valence', and ignore the band index. In this limit, H_C consists of gate-screened Coulomb interaction $V_C(\mathbf{q})$ projected onto the active valence bands. The numerical studies are carried out in the full 6-band (per spin, per valley) basis without resorting to band-projection.

Let us now consider the symmetries of H. It conserves both total electric charge and electron number in each valley, and thus possesses $\mathrm{U}(1)_c\times \mathrm{U}(1)_v$ symmetry. The spatial symmetries include translations by the Bravais lattice vectors $\boldsymbol{a}_{1/2} = a(1, \pm\sqrt{3})/2$ corresponding to the honeycomb lattice $T_{1/2}$ ($a = 0.246$ nm is the graphene lattice constant), a mirror M_x and C_3. At zero perpendicular displacement field, there is an additional inversion symmetry, forming the space group P$\bar{3}$m1 [3, 4], but inversion is broken for $u \neq 0$, which is required for observing correlated physics. The symmetry actions are given by:

$$\begin{aligned}
\mathrm{U}(1)_c : c_{\tau,s,\mathbf{k},\sigma} &\to e^{i\theta_c}c_{\tau,s,\mathbf{k},\sigma}, \quad \mathrm{U}(1)_v : c_{\tau,s,\mathbf{k},\sigma} \to e^{i\tau\theta_v}c_{\tau,s,\mathbf{k},\sigma}\\
T_j : (x,y) \to (x,y) + \boldsymbol{a}_j, &\quad c_{\tau,s,\mathbf{k},\sigma} \to e^{i\tau\mathbf{K}\cdot\boldsymbol{a}_j}c_{\tau,s,\mathbf{k},\sigma}, \text{ where } j = 1 \text{ or } 2\\
M_x : (x,y) \to (-x,y), &\quad c_{\tau,s,\mathbf{k},\sigma} \to c_{-\tau,s,M_x(\mathbf{k}),\sigma}, \text{ where } M_x(k_x,k_y) = (-k_x,k_y)\\
C_3 : x+iy \to e^{2\pi i/3}(x+iy), &\quad c_{\tau,s,\mathbf{k},\sigma} \to c_{\tau,s,C_3[\mathbf{k}],\sigma}
\end{aligned} \tag{5}$$

In Eq. (5), translations act as internal symmetries on the field operators $c_{\tau,s,\mathbf{k},\sigma}$, M_x preserves sublattice but flips valley and the x-component of momenta, and C_3, which preserves both valley and sublattice, is taken to be centered on the A_1/B_3 sites so that its only action on $c_{\tau,s,\mathbf{k},\sigma}$ is to rotate the momenta. Note that any other choice of rotation center for C_3 will an an overall phase for the spinor in the sublattice space. In particular, it does not act differently on the two active sublattices, that lie directly on top of each other, for any consistent choice of rotation center. The internal symmetries include anti-unitary time-reversal $\mathcal{T}$ and global spin-rotation $\mathrm{SU}(2)_s$. However, neglecting lattice-scale effects leads to an enhanced $\mathrm{SU}(2)_+\times \mathrm{SU}(2)_-$ symmetry, corresponding to individual spin-rotations in the valleys.

$$\begin{aligned}
\mathcal{T} : c_{\tau,s,\mathbf{k},\sigma} &\to (is_y)_{ss'}c_{-\tau,s',-\mathbf{k},\sigma}, \quad i \to -i\\
\mathrm{SU}(2)_\pm : c_{\pm,s,\mathbf{k},\sigma} &\to [e^{i\theta_\pm \hat{\mathbf{n}}_\pm\cdot\mathbf{s}}]_{ss'}c_{\pm,s',\mathbf{k},\sigma}
\end{aligned} \tag{6}$$

In Eq. (6), $\mathbf{s} = (s_x, s_y, s_z)$ denote the Pauli matrices in spin-space, and $(\theta, \hat{\mathbf{n}})_\pm$ correspond to the angle and axis of spin-rotations in each valley labeled $\tau = \pm$. Including lattice scale effects such as inter-valley electron scattering will reduce the spin-rotation to a global $\mathrm{SU}(2)_s$, corresponding to a single choice of $(\theta, \hat{\mathbf{n}})$ for both valleys. The absence of spin-orbit coupling in graphene allows us to define a anti-unitary spinless time-reversal $\tilde{\mathcal{T}}$ that preserves spin, but flips valley and momentum, i.e, acts as $\tau_x K$ and takes $\mathbf{k} \to -\mathbf{k}$. $\tilde{\mathcal{T}}$ relates the band-dispersion in the two valleys,

setting $\varepsilon_{n,\tau}(\mathbf{k}) = \varepsilon_{n,-\tau}(-\mathbf{k})$ (note that the dispersion is independent of spin). Further, demanding that the Bloch wave-functions in opposite valleys are related by time-reversal implies:

$$u_{n,\tau,s,\mathbf{k}} = u^*_{n,-\tau,s,-\mathbf{k}}, \quad [\lambda^{\tau,\tau'}_{\mathbf{q}}(\mathbf{k})]^* = \lambda^{-\tau,-\tau'}_{-\mathbf{q}}(-\mathbf{k}) \tag{7}$$

Eq. (7) relates the 'intra-valley' form factors from opposite valleys, and also 'inter-valley' form factors at different momenta, and will prove useful later when we look at the IVC and superconducting phases.

Supplementary Note 2. DETAILS OF SELF-CONSISTENT HARTREE-FOCK CALCULATIONS

In the Hartree-Fock calculation, we solve the self-consistent equations for Slater determinant states characterized by the one-electron covariance matrix $P^{ss'}_{\tau,\tau'}(\mathbf{k}) = \left\langle \psi^\dagger_{\tau,s,\mathbf{k}} \psi_{\tau',s',\mathbf{k}} \right\rangle$ using the formulation described in Ref. 5. In the following, we will only consider kinetic energy and intra-valley Coulomb scattering in the HF calculation such that the enhanced SU(2)$_+\times$ SU(2)$_-$ spin-rotation symmetry remains. The absence of spin-orbit coupling further decouples spin from all other degrees of freedom, which allows us to first block diagonalize the covariance matrix in the spin space such that $P^{ss'}_{\tau,\tau'}(\mathbf{k}) = P^{s}_{\tau,\tau'}(\mathbf{k})\delta_{s,s'}$. Then we use both the 'ODA' and 'EDIIS' algorithms to solve the self-consistency equation [6, 7].

HF typically over-estimates the exchange energy-gain in a metallic state since it neglects screening of the interaction by mobile electrons. To capture this screening effect, we consider a random phase approximation (RPA) correction by itinerant fermions [8]:

$$H_C = \frac{1}{2A}\sum_{\mathbf{q}} V_C(\mathbf{q}) : \rho(\mathbf{q})\rho(-\mathbf{q}) : \xrightarrow{\text{RPA}} \frac{1}{2A}\sum_{\mathbf{q}} V_{\text{RPA}}(\mathbf{q}) : \rho(\mathbf{q})\rho(-\mathbf{q}) : \ , \text{ where } V_{\text{RPA}}(\mathbf{q}) = \frac{V_C(\mathbf{q})}{1+\chi_{\rho\rho}(\mathbf{q})V_C(\mathbf{q})} \tag{8}$$

where $V_C(\mathbf{q}) = e^2 \tanh(qD)/(2\epsilon q)$ is the repulsive dual gate-screened Coulomb interaction, and $\chi_{\rho\rho}(\mathbf{q})$ is the static Lindhard response function. We have neglected the frequency dependence of the screening, and $\chi_{\rho\rho}(\mathbf{q}) = \chi_0(1 - c\mathbf{q}^2/k_F^2 + \cdots)$ for small q/k_F, where $-\chi_0 = -\partial_\mu n_e$ is simply the density of states at the Fermi surface at $T = 0$, and c is an O(1) constant that depends on Fermi surface details [8]. In the limit $q \gg D^{-1}$, we can define a Thomas-Fermi screening wavevector $q_{\text{TF}} = -\frac{e^2}{\epsilon}\chi_0$. For the purpose of the HF calculation, we only keep χ_0, and neglect further $\mathbf{q}$ dependence of $\chi_{\rho\rho}(\mathbf{q})$. To ensure convergence of the self-consistent calculation, we use the non-interacting density of states χ_0 with fourfold isospin degeneracy.

The numerical results shown in the paper are obtained with $\epsilon = 4.4$, gate distance $D = 50\,\text{nm}$, using the projected valance band per spin per valley on a 71×71 momentum grid, with UV momentum cutoff $0.085a^{-1}$. We take $u = 30\,\text{meV}$ in main text Fig. 2(a) showing the full competition among all candidate states. We also explored the phase diagram at $u = 20 - 40\,\text{meV}$ and confirmed the robustness of all our observations. In main text Fig. 2(b), we show the phase diagram at $u = 0 - 30\,\text{meV}$ only concerning the competition between the IVC state and the fully symmetric state. We take $\chi_0 = 0.16\,\text{eV}^{-1}$ per unit cell for all figures except for Supplementary Figure 4 where we explored the effect of changing χ_0.

Depending on which symmetries are explicitly enforced, we find several self-consistent solutions that can be grouped into four categories: (i) a 'half-metal', including a spin polarized (SP) state that breaks the global SU(2)$_s$ symmetry, a valley polarized (VP) state that breaks the spinless time-reversal $\tilde{\mathcal{T}}$, and a spin-valley locked (SVL) state that breaks both global SU(2)$_s$ and $\tilde{\mathcal{T}}$ but preserves their combination, (ii) a spin-singlet/triplet IVC "half-metal" that breaks U(1)$_v$ but preserves $\tilde{\mathcal{T}}$ and global SU(2)$_s$, (iii) a metal that breaks both global SU(2)$_s$ and $\tilde{\mathcal{T}}$, including a spin-valley polarized "quarter metal" (SVP) and a partially spin and valley-polarized (SP-v) state, (iv) a metallic IVC state that breaks both global SU(2)$_s$ and U(1)$_v$, including a spin polarized IVC "quarter metal" (SP-IVC) and a partially spin-polarized IVC (IVC-s) state. States within the latter two groups cannot be distinguished by symmetry, but they appear at very different hole doping. Close to SC1, the competitive candidates are SP, VP, SVL, spin-singlet/triplet IVC, SP-v, and IVC-s; and close to SC2, only SVP and SP-IVC are energetically competitive. Due to the enlarged SU(2)$_+\times$ SU(2)$_-$ symmetry of the Hamiltonian, states within the first two groups are degenerate, so we only plot one example within each group. We note that SP, VP, and SVL are not always fully polarized for weaker interaction strength, and will adjust population between two spin/valley sectors to minimize energy.

As shown in Fig. 2 in the main text, the precise energetic ordering of the phases and the reconstructed Fermi surface topology are sensitive to the interaction strength, which is mainly controlled by the density of states χ_0. However, across a wide parameter regime, SP/VP/SVL and spin-singlet/triplet IVC are close in energy and favored over a fully symmetric metal close to SC1, which is expected from the Fock energy gain. SP-v and IVC-s are even lower in energy close to the transition to fully symmetric metal. However, these two states are not observed in the experiment due to the Hund's coupling that is not included in the HF calculation. We will discuss the role Hund's coupling plays in

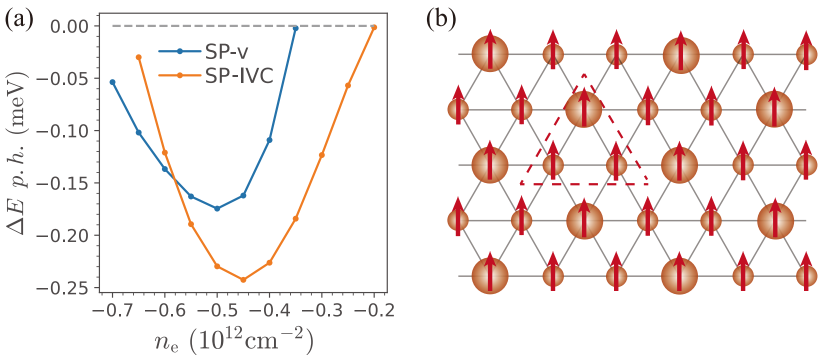

Supplementary Figure 2. (a) Hartree-Fock energetics assuming full spin-polarization at lower hole-doping, showing the competition between partially valley-polarized (SP-v) state and spin-polarized inter-valley coherent (SP-IVC) states, for parameters mentioned in the text. (b) Real space cartoon of the SP-IVC state, which is a ferromagnetic charge-density wave with a tripled unit cell (shown with dotted red lines).

this competition later in the SM. We also note that close to SC2, the SP-IVC phase (a ferromagnetic CDW in real space) can be energetically competitive with the SP-v phase (Supplementary Figure 2).

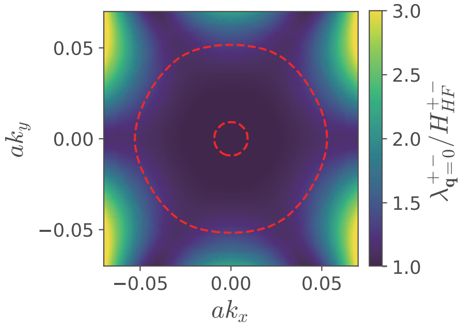

Supplementary Figure 3. The valley off-diagonal part of H_{HF} compared to the IVC density operator $n_S^{\rm IV}(\mathbf{q})$ at $\mathbf{q}=0$ in magnitude with proper normalization at $n_e = -1.05\times10^{12}\,\mathrm{cm}^{-2}$, which is almost uniform within the annular HF Fermi surface (indicated by dotted red lines).

Now we turn to analyze the structure of the self-consistent HF Hamiltonian H_{HF} deep in the IVC phase. In fact, the valley off-diagonal part of H_{HF} is very well approximated by the operator $n_{\rm S}^{\rm IV}(\mathbf{q})$ at $\mathbf{q}=\mathbf{0}$. In the momentum space, $n_{\rm S}^{\rm IV}(\mathbf{q})$ takes the form $\sum_{\mathbf{k},s}\lambda^{+-}_{\mathbf{q}}(\mathbf{k})\psi^\dagger_{+,s,\mathbf{k}}\psi_{-,s,\mathbf{k}+\mathbf{q}}$. We compared $\lambda^{+-}_{\mathbf{q}=\mathbf{0}}(\mathbf{k})$ with the valley off-diagonal part of HF Hamiltonian H^{+-}_{HF} at each $\mathbf{k}$, with proper normalization in Supplementary Figure 3. The fact that $\lambda^{+-}_{\mathbf{q}}/H^{+-}_{HF}$ is mostly uniform within the Fermi sea suggests that H^{+-}_{HF} captures a purely local $n_{\rm S}^{\rm IV}$ perturbation.

Finally, we comment on the effect of non-interacting density of states χ_0 used in the RPA screening. A smaller χ_0 can enhance the interaction and thus change the precise energetic ordering of the phases. When $\chi_0 = 0.08\,\mathrm{eV}^{-1}$, the SP/VP/SVL state and the spin singlet/triplet IVC state are much closer in energy and therefore their competition is almost entirely determined by the Hund's couple. In addition, we see that the phase transition toward an IVC phase becomes a second-order transition, which also leads to a divergent correlation length $\xi_{\rm IVC}$.

Supplementary Note 3. PERTURBATIVE HARTREE-FOCK ANALYSIS OF IVC ENERGETICS

To analyze the energetics of various isospin symmetry broken states analytically, we evaluate the energy of a general Slater determinant state characterized by a covariance matrix $P^{ss'}_{\tau,\tau'}(\mathbf{k}) = \left\langle \psi^\dagger_{\tau,s,\mathbf{k}}\psi_{\tau',s',\mathbf{k}}\right\rangle$. In this appendix, we will focus on two types of strong candidates close to SC1: (1) spin polarized (SP) state, valley polarized (VP) state, and spin-valley locked (SVL) state, and (2) spin-singlet/triplet IVC state. In the absence of Hund's coupling J_H, states within each group are degenerate within our Hartree-Fock analysis. Thus, we will only analyze the VP state and the spin-singlet IVC state, both of which are spin-singlet such that $P^{ss'}_{\tau,\tau'}(\mathbf{k}) = P_{\tau,\tau'}(\mathbf{k})\delta_{s,s'}$. Similar perturbative

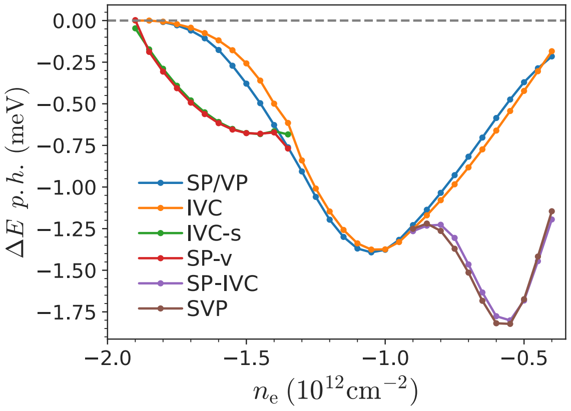

Supplementary Figure 4. Self-consistent HF energetics of isospin symmetry-broken states, including SP/VP/SVL, spin singlet/triplet IVC, SP-v, IVC-s, SVP, and SP-IVC using $\chi_0 = 0.08\,\mathrm{eV}^{-1}$ per unit cell, at $u = 30$ meV and $\epsilon = 4.4$. SC1 is observed approximately around $n_e = -1.9 \times 10^{12}\,\mathrm{cm}^{-2}$ and SC2 is observed around $n_e = -0.9 \times 10^{12}\,\mathrm{cm}^{-2}$.

Hartree-Fock analysis can also apply to states close to SC2, which includes the spin valley polarized (SVP) state and the spin-polarized IVC state.

The general Slater determinant state can be viewed as the ground state of a mean-field Hamiltonian H_{MF},

$$H_{MF} = \sum_{\tau,\boldsymbol{k}} \psi^\dagger_{\tau,\boldsymbol{k}} h^{MF}_{\tau,\tau'}(\boldsymbol{k}) \psi_{\tau',\boldsymbol{k}}, \quad \text{with } h^{MF}_{\tau,\tau'}(\boldsymbol{k}) = \begin{pmatrix} \varepsilon_\tau(\boldsymbol{k}) - \mu + \Delta_{VP}(\mathbf{k}) & \Delta^*_{IVC}(\boldsymbol{k}) \\ \Delta_{IVC}(\boldsymbol{k}) & \varepsilon_{-\tau}(\boldsymbol{k}) - \mu - \Delta_{VP}(\mathbf{k}) \end{pmatrix} \tag{9}$$

where $\Delta_{VP}(\mathbf{k})$ is the valley polarization, and $\Delta_{IVC}(\mathbf{k}) = |\Delta_{IVC}(\mathbf{k})| e^{i\phi_{\mathbf{k}}}$ is the IVC order parameter. Then the covariance matrix takes the form

$$P_{\tau,\tau'} = \left[\frac{1}{2}\left(1 + \frac{\boldsymbol{\Delta}^*_{\mathbf{k}} \cdot \boldsymbol{\tau}}{|\boldsymbol{\Delta}_{\mathbf{k}}|}\right) n_F(E_{\mathbf{k},+}) + \frac{1}{2}\left(1 - \frac{\boldsymbol{\Delta}^*_{\mathbf{k}} \cdot \boldsymbol{\tau}}{|\boldsymbol{\Delta}_{\mathbf{k}}|}\right) n_F(E_{\mathbf{k},-}) \right],$$
$$\text{where } \boldsymbol{\Delta}_{\mathbf{k}} \equiv (|\Delta_{IVC}(\mathbf{k})| \cos(\phi_{\mathbf{k}}), |\Delta_{IVC}(\mathbf{k})| \sin(\phi_{\mathbf{k}}), \xi_a(\mathbf{k}) + \Delta_{VP}(\mathbf{k})), \quad E_{\mathbf{k},\pm} = \xi_s(\mathbf{k}) \pm |\boldsymbol{\Delta}_{\mathbf{k}}|. \tag{10}$$

In Eq. (10), $\xi_s(\mathbf{k}) = (\varepsilon_{+,\mathbf{k}} + \varepsilon_{-,\mathbf{k}})/2 - \mu$ and $\xi_a(\mathbf{k}) = (\varepsilon_{+,\mathbf{k}} - \varepsilon_{-,\mathbf{k}})/2$ denote the valley-symmetric and valley-antisymmetric components of the dispersion respectively. Then we can evaluate the mean-field energy per spin using Wick's theorem,

$$\langle H \rangle_{MF} = \sum_{\boldsymbol{k},\tau} \xi_\tau(\boldsymbol{k}) P_{\tau\tau}(\boldsymbol{k}) + \frac{V_C(\mathbf{0}) N^2}{A} - \frac{1}{2A} \sum_{\mathbf{k},\boldsymbol{q}} V_C(\boldsymbol{q}) \lambda^{\tau\tau}_{\boldsymbol{q}}(\boldsymbol{k}) [\lambda^{\tau'\tau'}_{\boldsymbol{q}}(\boldsymbol{k})]^* P_{\tau\tau'}(\boldsymbol{k}) P_{\tau'\tau}(\boldsymbol{k}+\boldsymbol{q}) \tag{11}$$

The first term is the kinetic term, the second is the Hartree term which simply counts the total number of electrons per spin species $N = \sum_{\tau,k} P_{\tau\tau}(\boldsymbol{k})$ and the last term is the Fock term. Since the Hartree term does not distinguish different isospin symmetry broken states, we will neglect it from now on and consider only the other two terms. In the following, we will compare the Hartree-Fock energy of the VP state and the IVC state in two different limits.

A. Deep in the IVC phase

When the hole doping is low, only the lower mean field band is filled, then we can take $n_F(E_{\mathbf{k},+}) = 0$ for all $\mathbf{k}$. The kinetic energy becomes

$$\langle H_{kin} \rangle = \sum_{\tau,\mathbf{k}} \xi_\tau(\mathbf{k}) P_{\tau\tau}(\mathbf{k}) = \sum_{o.c.c} \xi_s(\mathbf{k}) - \sum_{o.c.c} \frac{\Delta^*_z(\mathbf{k})}{|\boldsymbol{\Delta}_{\mathbf{k}}|} \xi_a(\mathbf{k}) \tag{12}$$

We first define a reference state with dispersion $\xi_s(\mathbf{k})$ and work with a reference Fermi surface defined by $\xi_s(k_F(\theta)) = 0$ (Supplementary Figure 5(a)). In the following, we will fill this reference Fermi pocket for all isospin symmetry broken states instead of the Fermi pocket defined by the mean-field Hamiltonian in Eq.(9). We will add back Fermi surface

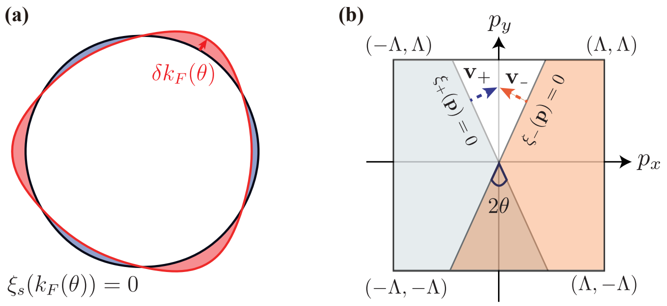

Supplementary Figure 5. (a) The black curve is the reference Fermi surface defined by $\xi_s(k_F(\theta)) = 0$. The red curve is the deformed Fermi surface $k_F(\theta) \to k_F(\theta) + \delta k_F(\theta)$ for a particular isospin symmetry broken state. (b) Intersecting Fermi surfaces of the two valleys at an angle 2θ (zoomed-in view with linearized dispersions). Filled regions are indicated by solid colors. $\mathbf{v}_\pm$ denote the Fermi velocities of the two valleys near the band-crossing point, and Λ is an upper momentum cutoff.

deformation δk_F from the reference Fermi surface perturbatively later. If we neglect Fermi surface deformation, the first term is the kinetic energy of the reference state and does not care about isospin symmetry breaking, so we will focus on the second term.

We start with two analytical limits: VP state with $\mathbf{\Delta}(\mathbf{k}) = \Delta_z(\mathbf{k})\hat{z}$ (with $\Delta_z(\mathbf{k}) > 0$) and IVC state with $\mathbf{\Delta}(\mathbf{k}) = \Delta_x(\mathbf{k})\hat{x} + \Delta_y(\mathbf{k})\hat{y}$. In these two limits, neither of them benefits from the kinetic energy term. For the IVC state, $\Delta_z^*(\mathbf{k}) = 0$ for all $\mathbf{k}$. For the VP state, the summation of $\xi_a(\mathbf{k})$ over occupied states gives zero due to time reversal symmetry. Now we discuss small perturbations to these two limits. For the VP state, if we introduce a small in-plane component $\mathbf{\Delta}(\mathbf{k}) = (\delta_x(\mathbf{k}), \delta_y(\mathbf{k}), \Delta_z(\mathbf{k}))$, leading order correction to the kinetic energy is to the second order in δ/Δ since $\Delta/\sqrt{\Delta^2 + \delta^2} \approx 1 - \delta^2/(2\Delta^2)$. However, a small local valley-polarization in the IVC state $\mathbf{\Delta}(\mathbf{k}) = (\Delta_x(\mathbf{k}), \Delta_y(\mathbf{k}), \delta_z(\mathbf{k}))$ results in a kinetic energy change which is linear in δ/Δ,

$$\Delta E_{kin}^{IVC} = -\sum_{|\mathbf{k}|<k_F(\theta)} \frac{\delta_z(\mathbf{k})}{|\Delta_\mathbf{k}|}\xi_a(\mathbf{k}) \tag{13}$$

which is negative if $\delta_z(\mathbf{k})$ follows the sign of the local valley-Zeeman field $B_{VZ}(\mathbf{k}) = 2\xi_a(\mathbf{k})$. Since $B_{VZ}(-\mathbf{k}) = -B_{VZ}(\mathbf{k})$, we can consistently choose $\delta_z(-\mathbf{k}) = -\delta_z(\mathbf{k})$, resulting in no net valley-polarization. This kinetic energy gain from valley-isospin vector canting is the major reason why the IVC state can be energetically competitive in spite of the interaction energy cost due to the opposite chirality of two valleys. It may seem that the kinetic energy gain is unbounded and the IVC state will automatically flow to the a fully symmetric metal such that $\mathbf{\Delta}(\mathbf{k}) = \xi_a(\mathbf{k})\hat{z}$. This does not happen because there is an interaction energy cost associated with local canting as we will show later.

We also note that the VP state gains kinetic energy through Fermi surface deformation $\delta k_F(\theta)$ (Supplementary Figure 5(a)).

$$\Delta E_{kin}^{VP} = \sum_{|\mathbf{k}|<k_F(\theta)+\delta k_F(\theta)} \xi_{-,\mathbf{k}} - \sum_{|\mathbf{k}|<k_F(\theta)} \xi_{-,\mathbf{k}} \approx -\left\{ \sum_{|\mathbf{k}|<k_F(\theta)+\delta k_F(\theta)} \xi_{a,\mathbf{k}} - \sum_{|\mathbf{k}|<k_F(\theta)} \xi_{a,\mathbf{k}} \right\} \tag{14}$$

where we take $\delta k_F(\theta + \pi) \approx -\delta k_F(\theta)$ to ensure that the area within the Fermi surface remains the same, and group terms at $\mathbf{k}$ and $-\mathbf{k}$ in the second equality. The final result corresponds a summation over shaded areas in Supplementary Figure 5(a), which contributes positively (negatively) in the red (blue) shaded regime. ΔE_{kin}^{VP} is negative if $\delta k_F(\theta)$ follows the sign of the local valley-Zeeman field $B_{VZ}(\mathbf{k})$. This can be intuitively understood as the kinetic energy gain by filling within the Fermi surface of the mean-field Hamiltonian defined by $\xi_-(k_F^-(\theta)) = 0$ instead of the reference Fermi surface. The actual Fermi surface would not deform all the way toward $k_F^-(\theta)$ since Fermi surface deformation has an associated interaction energy cost. However, it sets a hard upper limit on how much kinetic energy Fermi surface deformation can gain, which is achieved when the Fermi surface becomes the Fermi surface of the mean-field Hamiltonian, $k_F(\theta) + \delta k_F(\theta) = k_F^-(\theta)$. This is why when the interaction is sufficiently weak, the IVC state gains much more energy than the VP state of which the Fermi surface deformation has already saturated.

Now we turn to discuss the interaction (Fock) energy in this two limits. For the VP state, the covariance matrix takes a simple form

$$P_{\tau\tau'}(\boldsymbol{k}) = \begin{pmatrix} 0 & 0 \\ 0 & 1 \end{pmatrix} \tag{15}$$

in the $\{+,-\}$ basis. Then the Fock energy becomes

$$H_{Fock}^{VP} = -\frac{1}{2A}\sum_{\substack{\mathbf{k},\mathbf{q}\\ o.c.c.}} V_C(\boldsymbol{q})|\lambda_{\boldsymbol{q}}^{--}(\boldsymbol{k})|^2 \tag{16}$$

where the summation over $\mathbf{k}$ and $\mathbf{q}$ is performed with $\mathbf{k}$ and $\mathbf{k}+\mathbf{q}$ both inside the reference Fermi surface. We note that this is lower than what one would expect from a fully symmetric metal characterized by $P(\mathbf{k}) = \tau^0/2$,

$$\Delta E_{Fock}^{Stoner} = -\frac{1}{4A}\sum_{\substack{\mathbf{k},\mathbf{q}\\ o.c.c.}} V_C(\boldsymbol{q})|\lambda_{\boldsymbol{q}}^{--}(\boldsymbol{k})|^2 \tag{17}$$

which is the driving force of isospin polarization. The IVC state has a slightly more complicated covariance matrix structure,

$$P_{\tau\tau'}(\boldsymbol{k}) = \frac{1}{4}\begin{pmatrix} 1-\frac{\delta_z(\mathbf{k})}{|\Delta_{IVC}(\mathbf{k})|} & e^{i\phi_{\mathbf{k}}}\left(1-\frac{\delta_z^2(\mathbf{k})}{2|\Delta_{IVC}(\mathbf{k})|^2}\right) \\ e^{-i\phi_{\mathbf{k}}}\left(1-\frac{\delta_z^2(\mathbf{k})}{2|\Delta_{IVC}(\mathbf{k})|^2}\right) & 1+\frac{\delta_z(\mathbf{k})}{|\Delta_{IVC}(\mathbf{k})|}\end{pmatrix} \tag{18}$$

up to second order in δ/Δ. Then the Fock energy becomes

$$\begin{aligned} H_{Fock}^{IVC} = &-\frac{1}{4A}\sum_{\substack{\mathbf{k},\mathbf{q}\\ o.c.c.}} V_C(\boldsymbol{q})\Bigg\{|\lambda_{\boldsymbol{q}}^{++}(\boldsymbol{k})|^2\left(1+\frac{\delta_z(\mathbf{k})\delta_z(\mathbf{k}+\mathbf{q})}{|\Delta_{IVC}(\mathbf{k})|^2}\right) \\ &-\left(|\lambda_{\boldsymbol{q}}^{++}(\boldsymbol{k})|^2-|\lambda_{\boldsymbol{q}}^{--}(\boldsymbol{k})|^2\right)\frac{\delta_z(\mathbf{k})+\delta_z(\mathbf{k}+\mathbf{q})}{|\Delta_{IVC}(\mathbf{k})|} \\ &+|\lambda_{\boldsymbol{q}}^{++}(\boldsymbol{k})||\lambda_{\boldsymbol{q}}^{--}(\boldsymbol{k})|\left(1-\frac{\delta_z^2(\mathbf{k})+\delta_z^2(\mathbf{k}+\mathbf{q})}{2\,|\Delta_{IVC}(\mathbf{k})|^2}\right)\cos\left(\phi_{\boldsymbol{k}}-\phi_{\boldsymbol{k}+\boldsymbol{q}}+\boldsymbol{q}\cdot\left(\boldsymbol{A}_{\boldsymbol{k}}^{+}-\boldsymbol{A}_{\boldsymbol{k}}^{-}\right)\right)\Bigg\} \qquad (19) \\ \approx &-\frac{1}{2A}\sum_{\substack{\mathbf{k},\mathbf{q}\\ o.c.c.}} V_C(\boldsymbol{q})|\lambda_{\boldsymbol{q}}^{++}(\boldsymbol{k})|^2\left\{\left(1-\frac{q^2\,|\boldsymbol{\nabla}_{\boldsymbol{k}}\delta_z(\mathbf{k})|^2}{4\,|\Delta_{IVC}(\mathbf{k})|^2}\right)-\frac{q^2}{8}\left(\boldsymbol{\nabla}_{\boldsymbol{k}}\phi_{\boldsymbol{k}}-\left(\boldsymbol{A}_{\boldsymbol{k}}^{+}-\boldsymbol{A}_{\boldsymbol{k}}^{-}\right)\right)^2\right\} \qquad (20) \end{aligned}$$

where we used $\lambda_{\boldsymbol{q}}^{\tau\tau}(\boldsymbol{k}) \approx |\lambda_{\boldsymbol{q}}^{\tau\tau}(\boldsymbol{k})|e^{i\boldsymbol{q}\cdot\boldsymbol{A}_{\boldsymbol{k}}^{\tau}}$ in the first step (for small $|\mathbf{q}|$), where $\mathbf{A}^{\tau}(\mathbf{k}) = -i\langle u_{\tau,s,\mathbf{k}}|\nabla_{\mathbf{k}}u_{\tau,s,\mathbf{k}}\rangle$ is the Berry connection in valley $\tau = \pm$ [9]. In the second line, we further approximated $|\lambda_{\boldsymbol{q}}^{++}(\boldsymbol{k})| \approx |\lambda_{\boldsymbol{q}}^{--}(\boldsymbol{k})|$, Taylor expanded around small $\mathbf{q}$ and averaged over the dot product.

There are two additional energy cost compared to the VP state. One is the energy cost associated with the phase winding of the IVC order parameter due to opposite chirality in two valleys [5, 10–13],

$$\Delta E_{Fock}^{IVC,wind} = \frac{1}{16A}\sum_{\substack{\mathbf{k},\mathbf{q}\\ o.c.c.}} q^2 V_C(\boldsymbol{q})|\lambda_{\boldsymbol{q}}^{++}(\boldsymbol{k})|^2\left(\boldsymbol{\nabla}_{\boldsymbol{k}}\phi_{\boldsymbol{k}}-\left(\boldsymbol{A}_{\boldsymbol{k}}^{+}-\boldsymbol{A}_{\boldsymbol{k}}^{-}\right)\right)^2 \tag{21}$$

The other term is associated with canting of the valley isospin vector,

$$\Delta E_{Fock}^{IVC,cant} = \frac{1}{8A}\sum_{\substack{\mathbf{k},\mathbf{q}\\ o.c.c.}} V_C(\boldsymbol{q})q^2|\lambda_{\boldsymbol{q}}^{++}(\boldsymbol{k})|^2\frac{|\boldsymbol{\nabla}_{\boldsymbol{k}}\delta_z(\mathbf{k})|^2}{|\Delta_{IVC}^2(\mathbf{k})|^2} \tag{22}$$

Along with the kinetic energy gain ΔE_{kin}^{IVC}, it determines the local valley polarization $\delta_z(\mathbf{k})$ in the IVC state. Thus, the IVC state is energetically more favorable when the Coulomb repulsion is weak since the winding energy cost reduces and the kinetic energy gain increases due to a larger local valley polarization. This is precisely the case in RTG since the large density of states near the Fermi surface at low hole-doping can strongly screen the Coulomb repulsion.

B. Close to the onset of IVC phase

In this section, we carry out a complimentary analysis of the IVC kinetic energy using linearized band dispersions, by focusing on the crossing points of the valence bands from the two valleys that are gapped out by the development

of the IVC order. This applies when the order parameter magnitude is small. It has the advantage of being amenable to explicit analytical evaluation of the IVC kinetic energy including Fermi surface deformation effects, at the expense of introducing a cutoff momentum Λ away from beyond which the IVC order parameter vanishes.

To set up the problem, we consider the intersection points of the Fermi surfaces from the two valleys at an angle 2θ, as shown in Supplementary Figure 5(b). All symmetry-related crossings will have identical contributions to the energy, so it is sufficient to just focus on one crossing. We introduce a local coordinate system (p_x, p_y) centered at the crossing point, and linearize the band dispersions about this point:

$$\xi_\pm(\mathbf{p}) = \mathbf{v}_\pm \cdot \mathbf{p} = v(p_y \sin\theta \pm p_x \cos\theta) \tag{23}$$

We first establish that at a given filling, the chemical potential remains unchanged across the transition within this linear approximation. To do so, we need the mean-field spectrum of the symmetry-broken band structure, which in the most general case ($\Delta_{VP} \neq 0$ and $\Delta_{IVC} \neq 0$) is given by:

$$E_\pm(\mathbf{p}) = \xi_s(\mathbf{p}) \pm \sqrt{(\xi_a(\mathbf{p}) + \Delta_{VP})^2 + |\Delta_{IVC}(\mathbf{p})|^2}. \tag{24}$$

Within the linearized dispersion, we have:

$$\xi_s(\mathbf{p}) = \frac{\xi_{+,\mathbf{p}} + \xi_{-,\mathbf{p}}}{2} = vp_y \sin\theta, \text{ and } \xi_a(\mathbf{p}) = \frac{\xi_{+,\mathbf{p}} - \xi_{-,\mathbf{p}}}{2} = vp_x \cos\theta,$$
$$\text{and } E_\pm(\mathbf{p}) = vp_y \sin\theta \pm \sqrt{(vp_x\cos\theta + \Delta_{VP})^2 + |\Delta_{IVC}|^2} \tag{25}$$

where we have also assumed that we can neglect the $\mathbf{p}$ dependence of the IVC gap $|\Delta_{IVC}|$ near the crossing point. We note that $\xi_s(p_x, -p_y) = -\xi_s(p_x, p_y)$ and $\xi_a(p_x, -p_y) = \xi_a(p_x, p_y)$, such that $E_+(p_x, p_y) = -E_-(p_x, -p_y)$, indicating that the size of the hole Fermi pocket for the lower band (E_-) is the same as the size of the electron Fermi pocket of the upper band (E_+). More rigorously, we have (within a patch of size $2\Lambda \times 2\Lambda$ centered at the crossing point $\mathbf{p} = 0$):

$$\sum_{\mathbf{p}} \Theta(-E_-(\mathbf{p})) + \Theta(-E_+(\mathbf{p})) = \sum_{\mathbf{p}} \Theta(-E_-(p_x, p_y)) + \Theta(-E_+(p_x, -p_y)) = \sum_{\mathbf{p}} \Theta(-E_-(\mathbf{p})) + \Theta(E_-(\mathbf{p})) = \Lambda^2$$
$$= \sum_{\mathbf{p}} \Theta(-\xi_-(\mathbf{p})) + \Theta(-\xi_+(\mathbf{p})) \tag{26}$$

Thus, we have shown that the occupancy remains unchanged if we retain the same chemical potential, indicating that the chemical potential remains unchanged when $|\Delta_{IVC}| \neq 0$ and/or $\Delta_{VP} \neq 0$ (this continues to hold for $\Delta_{VP}(\mathbf{p}) \propto \xi_a(\mathbf{p})$ too, i.e, when there is local canting, within the linearized dispersion approximation).

Let us now take specialize to the IVC phase with no local or global valley polarization ($\Delta_{VP} = 0$). The covariance matrix for this phase is given by:

$$P_{\tau\tau}(\mathbf{p}) = \xi_s(\mathbf{p})(\Theta_{\mathbf{p},+} + \Theta_{\mathbf{p},-}) + \frac{\tau\xi_a(\mathbf{p})}{\sqrt{\xi_a^2(\mathbf{p}) + |\Delta_{IVC}(\mathbf{p})|^2}}(\Theta_{\mathbf{p},+} - \Theta_{\mathbf{p},-}), \quad \tau = \pm$$
$$\langle H_{kin}\rangle_{IVC} = \sum_{\mathbf{p},\tau} \xi_\tau(\mathbf{p}) P_{\tau\tau}(\mathbf{p}) = \sum_{\mathbf{k}} \xi_s(\mathbf{p})(\Theta_{\mathbf{p},+} + \Theta_{\mathbf{p},-}) + \frac{\xi_a^2(\mathbf{p})}{\sqrt{\xi_a^2(\mathbf{p}) + |\Delta_{IVC}(\mathbf{p})|^2}}(\Theta_{\mathbf{k},+} - \Theta_{\mathbf{k},-})$$
$$\text{where } E_\pm(\mathbf{k}) = \xi_s(\mathbf{k}) \pm \sqrt{(\xi_a^2(\mathbf{k}) + |\Delta_{IVC}(\mathbf{k})|^2} \tag{27}$$

It is instructive to compare the kinetic energy of the IVC state with the kinetic energy $\langle H_{kin}\rangle_0$ of the fully symmetric metal ($\Delta_{IVC}(\mathbf{k}) = 0 = \Delta_{VP}$), and that of the valley polarized metallic phase (VP) where $\Delta_{IVC}(\mathbf{k}) = 0$ but $\Delta_{VP} \neq 0$ in the basis of states with eigen-energies $E_\pm$ (i.e, we are no longer labeling the states by valley index τ, even when valley is a good quantum number).

$$\langle H_{kin}\rangle_0 = \sum_{\mathbf{p}} \xi_s(\mathbf{p})(\Theta_{\mathbf{p},+} + \Theta_{\mathbf{p},-}) + |\xi_a(\mathbf{p})|(\Theta_{\mathbf{p},+} - \Theta_{\mathbf{k},-}),\ E_\pm(\mathbf{p}) = \xi_s(\mathbf{p}) \pm |\xi_a(\mathbf{p})|$$
$$\langle H_{kin}\rangle_{VP} = \sum_{\mathbf{p}} \xi_s(\mathbf{p})(\Theta_{\mathbf{p},+} + \Theta_{\mathbf{p},-}) + \frac{\xi_a(\mathbf{p})(\xi_a(\mathbf{p}) + \Delta_{VP})}{|\xi_a(\mathbf{p}) + \Delta_{VP}|}(\Theta_{\mathbf{p},+} - \Theta_{\mathbf{p},-}),\ E_\pm(\mathbf{p}) = \xi_s(\mathbf{p}) \pm |\xi_a(\mathbf{p}) + \Delta_{VP}| \tag{28}$$

From Eq. (28), we note that the VP phase has a higher kinetic energy than the symmetry-preserving metal, as $(\Theta_{\mathbf{p},+} - \Theta_{\mathbf{p},-}) < 0$ is always true, and $|\xi_a(\mathbf{p})| > 0$ while $\xi_a(\mathbf{p})\text{sign}(\xi_a(\mathbf{p}) + \Delta_{VP})$ takes both positive and negative values. Further, comparing Eqs. (27) and (28), we note that the IVC can have a higher kinetic energy than the symmetry-preserving metal, so long as we neglect the difference in deformation of Fermi surfaces, and $|\xi_a(\mathbf{p})| \leq \sqrt{\xi_a^2(\mathbf{p}) + |\Delta_{IVC}(\mathbf{p})|^2}$. However, it is incorrect to neglect Fermi surface deformation, and it turns out that within

the linearized dispersion approximation in Eq. (23), we can evaluate the kinetic energy $\langle H_{kin}\rangle$ of each of these states analytically. These results are presented below for different states (for a single patch, for the whole BZ we have to multiply by the appropriate number of symmetry related crossing points), along series expansions to lowest non-trivial order in $\Delta^2_{VP/IVC}$.

$$
\begin{aligned}
\langle H_{kin}\rangle_0 &= -2v\Lambda^3\left(\frac{\sin^2\theta}{3\cos\theta}+\cos\theta\right)\\
\langle H_{kin}\rangle_{VP} &= -2v\Lambda^3\left(\frac{\sin^2\theta}{3\cos\theta}+\cos\theta\right)+\frac{2\Lambda\Delta^2_{VP}}{v\cos\theta}=\langle H_{kin}\rangle_0+\frac{2\Lambda\Delta^2_{VP}}{v\cos\theta}\\
\langle H_{kin}\rangle_{IVC} &= -\frac{2v\sin^2\theta}{3\cos\theta}\left(\Lambda^2-\frac{\Delta^2_{IVC}}{v^2\sin^2\theta}\right)^{3/2}-2v\cos\theta\Lambda^2\left(\Lambda^2+\frac{\Delta^2_{IVC}}{v^2\cos^2\theta}\right)^{1/2}\\
&\quad+\frac{2\Delta^2_{VC}}{v\cos\theta}\left[\left(\Lambda^2-\frac{\Delta^2_{IVC}}{v^2\sin^2\theta}\right)^{1/2}-\Lambda\coth^{-1}\left(\frac{\Lambda}{\sqrt{\Lambda^2-\frac{\Delta^2_{IVC}}{v^2\sin^2\theta}}}\right)+\Lambda\tanh^{-1}\left(\frac{\Lambda}{\sqrt{\Lambda^2+\frac{\Delta^2_{IVC}}{v^2\cos^2\theta}}}\right)\right]\\
&\approx -2v\Lambda^3\left(\frac{\sin^2\theta}{3\cos\theta}+\cos\theta\right)+\frac{2\Lambda\Delta^2_{IVC}}{v\cos\theta}\left(1+\ln(\cot\theta)\right)\\
&= \langle H_{kin}\rangle_0+\frac{2\Lambda\Delta^2_{IVC}}{v\cos\theta}\left(1+\ln(\cot\theta)\right)
\end{aligned}
\tag{29}
$$

We note that the IVC state tends pay more kinetic energy penalty as $\theta\to 0$ or $\theta\to\pi/2$, corresponding to $2\theta=0$ or π, i.e, perfect nesting of the Fermi surfaces from the two valleys in the patch considered. Further, generally for same magnitude of gap, $\langle H_{kin}\rangle_{IVC}>\langle H_{kin}\rangle_{VP}$, although this should not be taken very seriously as the UV cutoff imposed is somewhat arbitrary and the IVC order parameter Δ_{IVC} does depend on $\mathbf{p}$ (it is only approximately constant close to the band-crossing points).

However, the kinetic energy term in the IVC state stands to gain when we introduce a momentum dependent valley polarization $\Delta_{VP}(\mathbf{p})\propto\xi_a(\mathbf{p})$ (valley-antisymmetric part of dispersion). While this doesn't lead to an overall valley polarization as time-reversal symmetry of the single-particle band structure implies that $\langle\Delta_{VP}(\mathbf{p})\rangle=0$ averaged over a Fermi pocket, it aids the kinetic term. This can be seen explicitly by using a mean-field ansatz with $\Delta_{VP}(\mathbf{p})=(\alpha-1)\xi_a(\mathbf{k})$, where α is a control parameter that tunes the degree of canting ($\alpha=1$ implies no canting). For such a state, we note that $E_\pm(\mathbf{p})=\xi_s(\mathbf{p})\pm\sqrt{(\alpha\xi_a(\mathbf{p}))^2+|\Delta_{IVC}(\mathbf{p})|^2}$. Consequently,

$$
\begin{aligned}
P_{\tau\tau}(\mathbf{k}) &= \xi_s(\mathbf{p})(\Theta_{\mathbf{p},+}+\Theta_{\mathbf{p},-})+\frac{\tau\alpha\xi_a(\mathbf{p})}{\sqrt{(\alpha\xi_a(\mathbf{p}))^2+|\Delta_{IVC}(\mathbf{p})|^2}}(\Theta_{\mathbf{p},+}-\Theta_{\mathbf{p},-}),\quad \tau=\pm\\
\langle H_{kin}\rangle &= \sum_{\mathbf{p},\tau}\xi_\tau(\mathbf{p})P_{\tau\tau}(\mathbf{p})=\sum_{\mathbf{p}}\xi_s(\mathbf{p})(\Theta_{\mathbf{p},+}+\Theta_{\mathbf{p},-})+\frac{\alpha\xi_a^2(\mathbf{p})}{\sqrt{(\alpha\xi_a(\mathbf{p}))^2+|\Delta_{IVC}(\mathbf{p})|^2}}(\Theta_{\mathbf{p},+}-\Theta_{\mathbf{p},-})
\end{aligned}
\tag{30}
$$

We again approximate $\Delta_{IVC}(\mathbf{p})\approx\Delta_{IVC}$ near the band-crossing points, and linearize the band dispersions. By our previous arguments, the chemical potential remains unchanged for such an ansatz, and the kinetic energy can be calculated explicitly.

$$
\begin{aligned}
\langle H_{kin}\rangle &= \left(\Lambda^2-\frac{\Delta^2_{IVC}}{v^2\sin^2\theta}\right)^{3/2}\left[-\frac{4v\sin^2\theta}{3\alpha\cos\theta}+\frac{2v\sin^2\theta}{3\alpha^2\cos\theta}\right]-2v\cos\theta\Lambda^2\left(\Lambda^2+\frac{\Delta^2_{IVC}}{\alpha^2v^2\cos^2\theta}\right)^{1/2}\\
&\quad+\frac{2\Delta^2_{IVC}}{\alpha^2v\cos\theta}\left[\left(\Lambda^2-\frac{\Delta^2_{IVC}}{v^2\sin^2\theta}\right)^{1/2}-\Lambda\coth^{-1}\left(\frac{\Lambda}{\sqrt{\Lambda^2-\frac{\Delta^2_{IVC}}{v^2\sin^2\theta}}}\right)+\Lambda\tanh^{-1}\left(\frac{\Lambda}{\sqrt{\Lambda^2+\frac{\Delta^2_{IVC}}{\alpha^2v^2\cos^2\theta}}}\right)\right]\\
&\approx -\frac{2v\Lambda^3}{3v\cos\theta}\left[\left(\frac{2}{\alpha}-\frac{1}{\alpha^2}\right)\sin^2\theta+3\cos^2\theta\right]+\frac{2\Delta^2_{IVC}}{\alpha^2v\cos\theta}\left[\frac{1}{\alpha}+\frac{1}{\alpha^2}\ln(\alpha\cot\theta)\right]
\end{aligned}
\tag{31}
$$

First, we note that the the $\alpha=1$ limit recovers the IVC kinetic energy in Eq. (29), which acts as a sanity check. Next, we note that if we write $\alpha=1+\delta_Z$ and expand in small δ_Z, there are corrections to both linear and quadratic orders in δ_Z:

$$
\langle H_{kin}\rangle=\langle H_{kin}\rangle_{IVC}+\frac{2v\Lambda^3}{3\cos\theta}\delta_Z^2+\frac{2\Delta^2_{IVC}}{v\cos\theta}\left[-2\ln(\cot\theta)\delta_Z+3\left(\ln(\cot\theta)-\frac{1}{2}\right)\delta_Z^2\right]
\tag{32}
$$

Roughly speaking, the linear correction arises from the term involving the valley-symmetric part of the dispersion $\sum_{\mathbf{p}} \xi_s(\mathbf{p})(\Theta_{\mathbf{p},+} + \Theta_{\mathbf{p},-})$. While $\xi_s(\mathbf{p})$ itself does not change, the Fermi surface occupancies change, resulting in a linear correction in δ_Z to the lowest order. The quadratic correction arises from the next higher order correction to the symmetric part, as well as to leading order from the valley-antisymmetric part of dispersion, as $\Delta_{VP}(\mathbf{p})$ directly adds on to $\xi_a(\mathbf{p})$. This implies that the kinetic energy for a given non-zero Δ_{IVC} cannot have a minima at $\delta_Z = 0$, and thus such a minima will always be shifted to $\delta_Z \neq 0$. Thus, the kinetic energy of the IVC state can always be lowered by canting, i.e, by having some $\Delta_{VP}(\mathbf{p}) \propto \xi_a(\mathbf{p})$ near the Fermi surface. This is exactly what is observed in Fig. 2(c) in the main text.

Supplementary Note 4. DERIVATION OF INTER-VALLEY HUND'S COUPLING

The inter-valley Hund's coupling plays a crucial role in determining the phases of ABC graphene in presence of a displacement field, as discussed above. Therefore, in this section, we work out the inter-valley Hund's coupling term for RTG microscopically by including an arbitrary translation invariant density-density interaction $U(\mathbf{r}, \mathbf{r}') = U(\mathbf{r}-\mathbf{r}')$ in real space. The following interaction Hamiltonian is our starting point (: : denotes normal ordering):

$$H_I = \frac{1}{2} \sum_{\mathbf{r},\mathbf{r}'} U(\mathbf{r}, \mathbf{r}') : \rho(\mathbf{r})\rho(\mathbf{r}') : \tag{33}$$

Each lattice site $\mathbf{r}$ can be labeled by a Bravais lattice position $\mathbf{R}$ of the two-dimensional unit cell, together with a basis position index $a \in \{A_1, B_1, A_2, B_2, A_3, B_3\}$ which determines both sublattice and layer (σ in previous/later sections denote a subset of these indices relevant for active bands near charge neutrality). Now, we note that the local electron operator can be written in terms of the monolayer low-energy Dirac fermions $c_{\tau,s,\mathbf{k},a}$ as (N = number of unit cells):

$$c_{\mathbf{R},a,s} = \frac{1}{\sqrt{N}} \sum_{\mathbf{k},\tau} e^{i(\mathbf{k}+\tau\mathbf{K})\cdot\mathbf{R}_a} c_{\tau,s,\mathbf{k},a} \tag{34}$$

The summation over $\mathbf{k}$ is implicitly restricted to $|\mathbf{k}| \leq \Lambda_{UV}$, where Λ_{UV} is some ultra-violet cutoff on the scale of the lattice spacing, that also satisfies $\Lambda_{UV} \ll |\mathbf{K}|$. This implies that the density operator takes the following form:

$$\rho(\mathbf{r}) = \rho(\mathbf{R}, a) = \sum_s c^\dagger_{\mathbf{R},a,s} c_{\mathbf{R},a,s} = \frac{1}{N} \sum_{\mathbf{k},\mathbf{q},\tau,\tilde{\tau},s} e^{i[\mathbf{q}+(\tilde{\tau}-\tau)\mathbf{K}]\cdot\mathbf{R}_a} c^\dagger_{\tau,s,\mathbf{k},a} c_{\tilde{\tau},s,\mathbf{k}+\mathbf{q},a} \equiv \frac{1}{N} \sum_{\mathbf{q}} e^{i\mathbf{q}\cdot\mathbf{R}_a} \rho_a(\mathbf{q}) \tag{35}$$

where by the last equality we have have defined the electron density $\rho_a(\mathbf{q})$ in momentum space. Note that it has a slowly varying component which modulates at momenta $|\mathbf{q}|$ (when $\tilde{\tau} = \tau$), and a fast-varying component that modulates at momenta $\mathbf{q} \pm 2\mathbf{K}$ for $\tilde{\tau} = -\tau$. While only the slowly varying component of density was considered in the interaction term for our Hartree-Fock numerics, here we keep both terms. Since we want to study the effect of this interaction projected to the 'active' low-energy bands, we re-write the valence-band projected density operator in the Bloch or band-basis of RTG:

$$\rho_a(\mathbf{q}) = \sum_{\mathbf{k},s} \sum_{\tau,\tilde{\tau}} \lambda^{\tau,\tilde{\tau}}_{a,\mathbf{q}}(\mathbf{k}) e^{i[\mathbf{q}+(\tilde{\tau}-\tau)\mathbf{K}]\cdot\mathbf{R}_a} \psi^\dagger_{\tau,s,\mathbf{k}} \psi_{\tilde{\tau},s,\mathbf{k}+\mathbf{q}} \tag{36}$$

where have restricted to the valence band above and neglected the n index. $\lambda^{\tau,\tilde{\tau}}_{a,\mathbf{q}}(\mathbf{k})$ is the sublattice and layer projected form-factor, given by:

$$\lambda^{\tau,\tilde{\tau}}_{a,\mathbf{q}}(\mathbf{k}) = \langle u_{\tau,\mathbf{k}}| P_a |u_{\tilde{\tau},\mathbf{k}+\mathbf{q}}\rangle \tag{37}$$

For a translation invariant potential $U(\mathbf{r}-\mathbf{r}')$ with $\mathbf{r} = (\mathbf{R}, a)$ and $\mathbf{r}' = (\mathbf{R}', b)$, we can define a Fourier transform as:

$$U(\mathbf{r}, \mathbf{r}') = U(\mathbf{r}-\mathbf{r}') = \frac{1}{A} \sum_{\mathbf{p}} U_{ab}(\mathbf{p}) e^{-i\mathbf{p}\cdot(\mathbf{R}_a - \mathbf{R}'_b)} \tag{38}$$

Plugging this into Eq. (33) and carrying out the summations over the lattice positions, we find that overall momentum conservation leads to net valley-charge conservation (in absence of Umklapp processes which doesn't appear at this order [14]). Specifically, the interaction term takes the form:

$$H_I = \frac{1}{2A} \sum_{\mathbf{k},s,\mathbf{k}',s'} \sum_{\tau,\tilde{\tau}} U_{ab}(\mathbf{p}) \lambda^{\tau,\tilde{\tau}}_{a,\mathbf{q}}(\mathbf{k}) \lambda^{\tau',\tilde{\tau}'}_{b,-\mathbf{q}}(\mathbf{k}') : \psi^\dagger_{\tau,s,\mathbf{k}} \psi_{\tilde{\tau},s,\mathbf{k}+\mathbf{q}} \psi^\dagger_{\tau',s,\mathbf{k}'} \psi_{\tilde{\tau}',s,\mathbf{k}'-\mathbf{q}} :$$

$$\times \left(\frac{1}{N} \sum_{\mathbf{R}_a} e^{i[-\mathbf{p}+\mathbf{q}+(\tilde{\tau}-\tau)\mathbf{K}]\cdot\mathbf{R}_a} \right) \left(\frac{1}{N} \sum_{\mathbf{R}'_b} e^{i[\mathbf{p}-\mathbf{q}+(\tilde{\tau}'-\tau')\mathbf{K}]\cdot\mathbf{R}'_b} \right) \tag{39}$$

where the summation over $\mathbf{R}_a$ and $\mathbf{R}'_b$ gives:

$$\mathbf{p} = \mathbf{q} + (\tilde{\tau} - \tau)\mathbf{K} = \mathbf{q} - (\tilde{\tau'} - \tau')\mathbf{K} \implies (\tau - \tau' + \tilde{\tau} - \tilde{\tau'})\mathbf{K} = 0 \bmod \mathbf{G} \tag{40}$$

where $\mathbf{G}$ is any reciprocal lattice vector. Since $3\mathbf{K}$ satisfies this condition but not $2\mathbf{K}$, we are only left with two options: (i) $\tau = \tau', \tilde{\tau} = \tilde{\tau'}$ which corresponds to intra-valley scattering terms with small momentum transfer $|\mathbf{p}|$, such terms respect $\mathrm{SU}(2)_+ \times \mathrm{SU}(2)_-$, and (ii) $\tau = -\tau' = -\tilde{\tau} = \tilde{\tau'}$ which correspond to terms which scatter between valleys, such terms break $\mathrm{SU}(2)_+ \times \mathrm{SU}(2)_-$ to a global $\mathrm{SU}(2)_s$ and give rise to Hund's. Type (i) terms allow for small momenta scattering, and are more important for long-range Coulomb interactions which decay at large momenta as $1/q$. Type (ii) terms necessarily involve momentum transfer of $|\mathbf{p}| \approx 2|\mathbf{K}|$, and therefore are more important for short-range electron-electron interactions (such as on-site Hubbard U). Thus, we arrive at the following form for the inter-valley interaction:

$$H_{\mathrm{inter-valley}} = \frac{1}{2A} \sum_{\mathbf{k},\mathbf{k}',\mathbf{q},\tau} \sum_{s,s'} \sum_{a,b} \left[U_{ab}(\mathbf{q} + 2\tau\mathbf{K}) \lambda^{\tau,-\tau}_{a,\mathbf{q}}(\mathbf{k}) \lambda^{-\tau,\tau}_{b,-\mathbf{q}}(\mathbf{k}') \right] : \psi^\dagger_{-\tau,s,\mathbf{k}} \psi_{\tau,s,\mathbf{k}+\mathbf{q}} \psi^\dagger_{\tau,s',\mathbf{k}'} \psi_{-\tau,s',\mathbf{k}'-\mathbf{q}} : \tag{41}$$

To derive the Hund's coupling, we use the Fierz identity $2\delta_{\alpha\nu}\delta_{\beta\mu} = \mathbf{s}_{\alpha\beta} \cdot \mathbf{s}_{\mu\nu} + \delta_{\alpha\beta}\delta_{\mu\nu}$ on the inter-valley scattering term derived in Eq. (41).

$$\begin{aligned} \sum_{s,s'} : \psi^\dagger_{-\tau,s,\mathbf{k}} \psi_{\tau,s,\mathbf{k}+\mathbf{q}} \psi^\dagger_{\tau,s',\mathbf{k}'} \psi_{-\tau,s',\mathbf{k}'-\mathbf{q}} : \quad &= \sum_{\alpha,\beta,\mu,\nu} : \psi^\dagger_{-\tau,\alpha,\mathbf{k}} \psi_{\tau,\beta,\mathbf{k}+\mathbf{q}} \psi^\dagger_{\tau,\mu,\mathbf{k}'} \psi_{-\tau,\nu,\mathbf{k}'-\mathbf{q}} : \ \delta_{\alpha\beta}\delta_{\mu\nu} \\ = -\sum_{\alpha,\beta,\mu,\nu} : (\psi^\dagger_{-\tau,\alpha,\mathbf{k}} \mathbf{s}_{\alpha\beta} \psi_{\tau,\beta,\mathbf{k}+\mathbf{q}}) \cdot (\psi^\dagger_{\tau,\mu,\mathbf{k}'} \mathbf{s}_{\mu\nu} \psi_{-\tau,\nu,\mathbf{k}'-\mathbf{q}}) : &+ 2\sum_{\alpha,\beta} : \psi^\dagger_{-\tau,\alpha,\mathbf{k}} \psi_{\tau,\beta,\mathbf{k}+\mathbf{q}} \psi^\dagger_{\tau,\beta,\mathbf{k}'} \psi_{-\tau,\alpha,\mathbf{k}'-\mathbf{q}} : \end{aligned} \tag{42}$$

Note that the second term in Eq. (42) is $\mathrm{SU}(2)_+ \times \mathrm{SU}(2)_-$ symmetric, while the first term has only global SU(2) symmetry. Defining a gauge-invariant (inter-valley) site-projected spin-operator as:

$$\mathbf{s}_{+-,a}(\mathbf{q}) = \sum_{\mathbf{k}} \lambda^{+-}_{a,\mathbf{q}}(\mathbf{k}) \psi^\dagger_{+,\alpha,\mathbf{k}} \mathbf{s}_{\alpha\beta} \psi_{-,\beta,\mathbf{k}+\mathbf{q}} \tag{43}$$

we see that the interaction Hamiltonian can be re-written in a particularly simple manner as:

$$H_{\mathrm{Hund's}} = -\frac{1}{2A} \sum_{\mathbf{q},a,b} U_{ab}(\mathbf{q}) \left[\mathbf{s}_{+-,a}(\mathbf{q}) \cdot \mathbf{s}_{-+,b}(-\mathbf{q}) + \mathbf{s}_{-+,a}(\mathbf{q}) \cdot \mathbf{s}_{+-,b}(-\mathbf{q}) \right] \tag{44}$$

This takes a particularly simple form when $U_{\mathbf{R}_a,\mathbf{R}'_b} = \tilde{U}\delta_{\mathbf{R},\mathbf{R}'}$, i.e, there is short-range interaction which is equal on all sublattice sites within the unit cell. While this is not physically accurate because of interlayer separation being much larger than intralayer separation, it is nevertheless useful for illustrating the basic physics. In this limit, we have $U_{ab}(\mathbf{q}) = (\sqrt{3}a^2/2)\tilde{U} \equiv U$, and therefore:

$$H_{\mathrm{Hund's}} = -\frac{U}{A} \sum_{\mathbf{q}} \mathbf{s}_{+-}(\mathbf{q}) \cdot \mathbf{s}_{-+}(-\mathbf{q}) = -\frac{U}{A} \sum_{\mathbf{q}} \mathbf{s}_{+-}(\mathbf{q}) \cdot \mathbf{s}^\dagger_{+-}(\mathbf{q}), \ \text{where } \mathbf{s}_{\tau,-\tau}(\mathbf{q}) = \sum_a \mathbf{s}_{\tau,-\tau,a}(\mathbf{q}) \tag{45}$$

which is Eq. (5) in the main text. Note that the magnitude of J_H is suppressed for long-range Coulomb interactions by a factor of $k_F/|\mathbf{K}| \ll 1$, k_F being the typical Fermi momentum which is small near charge neutrality. However, it is not necessarily small for short-range e-e scattering, and can thus contribute significantly to the choice of preferred ground state when iso-spin symmetry is broken.

We comment that form of coupling in Eq. (45) is quite different from the usual Hund's coupling (taken to be of the form $\mathbf{s}_+ \cdot \mathbf{s}_-$), familiar in the context of quantum hall ferromagnets and phenomenologically introduced in Ref. 2 to explain the flavor polarization observed in experiments. As we argued in the main text, the data in Refs. 2 and 15 can also be explained using the $H_{\mathrm{Hund's}}$ microscopically derived in Eq. (45). Because of the valley-crossed form-factors, after projection to the low-energy bands there is no Fierz transformation which can convert it to the form:

$$\tilde{H}_{\mathrm{Hund's}} = -\frac{\tilde{J}_H}{A} \sum_{\mathbf{q}} \mathbf{s}_+(\mathbf{q}) \cdot \mathbf{s}_-(-\mathbf{q}), \ \text{where } \mathbf{s}_\tau(\mathbf{q}) = \sum_{\mathbf{k}} \lambda^{\tau,\tau}_{\mathbf{q}}(\mathbf{k}) \psi^\dagger_{\tau,\alpha,\mathbf{k}} \mathbf{s}_{\alpha\beta} \psi_{\tau,\beta,\mathbf{k}+\mathbf{q}} \tag{46}$$

Thus, while the Hund's coupling in Eq. (46) is symmetry-allowed, any inter-valley electron-electron scattering, whether mediated by local interactions or by phonons, would not give rise to such a term (at least within a hopping model which neglects the orbital overlaps on different sites). For general $\mathbf{q}$ and $\mathbf{k}$, the opposite Berry-curvature of the two valleys imply that $\lambda^{\tau,\tau}_{\mathbf{q}}(\mathbf{k}) \neq \lambda^{\tau,-\tau}_{\mathbf{q}}(\mathbf{k})$. However, when the wave-functions are polarized to a single sublattice (say A_1), the distinction between $\lambda^{\tau,\tau}_{\mathbf{q}}(\mathbf{k})$ and $\lambda^{\tau,-\tau}_{\mathbf{q}}(\mathbf{k})$ vanishes, as these are both equal to one.

To summarize, there are two kinds of symmetry-allowed Hund's coupling, which can be written as follows by allowing for more general non-local interactions.

$$H_{\mathrm{Hund's}} = -\frac{1}{A}\sum_{\mathbf{q}} J_H(\mathbf{q})\ \mathbf{s}_{+-}(\mathbf{q})\cdot\mathbf{s}_{-+}(-\mathbf{q}), \tag{47}$$

$$\tilde{H}_{\mathrm{Hund's}} = -\frac{1}{A}\sum_{\mathbf{q}} \tilde{J}_H(\mathbf{q})\ \mathbf{s}_{+}(\mathbf{q})\cdot\mathbf{s}_{-}(-\mathbf{q}), \tag{48}$$

$H_{\mathrm{Hund's}}$ and $\tilde{H}_{\mathrm{Hund's}}$ are not related by Fierz transformations for Pauli matrices, unlike in a $\nu = 0$ graphene QH system [16], because of the presence of different form-factors arising from valley-projection. The only exception is the sublattice polarized limit (large displacement field or small kinetic energy), when the form-factors become trivial ($\lambda^{\tau,\tau'}_{\mathbf{q}}(\mathbf{k}) \approx 1$) and and the two Hamiltonians are equivalent upto $\mathrm{SU}(2)_+\times\mathrm{SU}(2)_-$ symmetric terms.

Supplementary Note 5. EFFECT OF HUNDS COUPLING ON ISO-SPIN SYMMETRY BROKEN STATES

In this section, we consider the perturbative effect of Hund's coupling on the different isospin symmetry-broken states. In particular, we derive the Hund's coupling induced splitting of the U(2) IVC manifold, relevant to the PIP phase near SC1. Simultaneously, we also discuss its effect on the spin-polarized IVC state, a possible candidate for the PIP phase near SC2. Finally, we also consider how inter-valley scattering can break the degeneracy between spin and valley polarization.

A. IVC states

First, we consider the Hund's coupling $H_{\mathrm{Hund's}}$ derived from microscopic considerations in Eq. (45). We first evaluate its expectation value in a general mean-field state, which is a Slater determinant characterized by a covariance matrix $P^{ss'}_{\tau,\tau'}(\mathbf{k}) = \langle \psi^\dagger_{\tau,s,\mathbf{k}}\psi_{\tau',s',\mathbf{k}}\rangle$, and then apply it to the different IVC states on interest (traces indicate tracing over spin indices, sum on index $i = x, y, z$ for Pauli matrices s^i is implicit):

$$\begin{aligned}
\langle H_{\mathrm{Hund's}}\rangle &= -\frac{1}{A}\sum_{\mathbf{q},\mathbf{k},\mathbf{k}'} J_H(\mathbf{q})\lambda^{+-}_{\mathbf{q}}(\mathbf{k})\lambda^{-+}_{-\mathbf{q}}(\mathbf{k}')s^i_{\alpha\beta}s^i_{\mu\nu}\langle\psi^\dagger_{+,\alpha,\mathbf{k}}\psi_{-,\beta,\mathbf{k}+\mathbf{q}}\psi^\dagger_{-,\mu,\mathbf{k}'}\psi_{+,\nu,\mathbf{k}'-\mathbf{q}}\rangle_{\mathrm{IVC}}\\
&= -\frac{1}{A}\sum_{\mathbf{k},\mathbf{k}'} J_H(\mathbf{0})\lambda^{+-}_{\mathbf{q}=\mathbf{0}}(\mathbf{k})\lambda^{-+}_{\mathbf{q}=\mathbf{0}}(\mathbf{k}')(\mathrm{Tr}\left[P_{+-}(\mathbf{k})(s^i)^T\right])(\mathrm{Tr}\left[P_{-+}(\mathbf{k}')(s^i)^T\right])\\
&\quad+\frac{1}{A}\sum_{\mathbf{q},\mathbf{k}} J_H(\mathbf{q})\lambda^{+-}_{\mathbf{q}}(\mathbf{k})\lambda^{-+}_{-\mathbf{q}}(\mathbf{k}+\mathbf{q})\ \mathrm{Tr}\left[P_{++}(\mathbf{k})(s^i)^T P_{--}(\mathbf{k}+\mathbf{q})(s^i)^T\right]\\
&= -\frac{J_H(\mathbf{0})}{A}\left(\sum_{\mathbf{k}}\lambda^{+-}_{\mathbf{q}=\mathbf{0}}(\mathbf{k})\ \mathrm{Tr}\left[P_{+-}(\mathbf{k})(s^i)^T\right]\right)\left(\sum_{\mathbf{k}'}\lambda^{-+}_{\mathbf{q}=\mathbf{0}}(\mathbf{k}')\ \mathrm{Tr}\left[P_{-+}(\mathbf{k}')(s^i)^T\right]\right)\\
&\quad+\frac{1}{A}\sum_{\mathbf{q},\mathbf{k}} J_H(\mathbf{q})|\lambda^{+-}_{\mathbf{q}}(\mathbf{k})|^2\ \mathrm{Tr}\left[P_{++}(\mathbf{k})(s^i)^T P_{--}(\mathbf{k}+\mathbf{q})(s^i)^T\right]
\end{aligned} \tag{49}$$

(i) Spin-singlet CDW IVC: For this state, the U(2) matrix $U_{ss'} = \delta_{s,s'}$, upto an overall phase. Using this, we find that the covariance matrix takes the form:

$$\begin{aligned}
P^{s,s'}_{\tau,\tau'}(\mathbf{k}) &= \left[\frac{1}{2}\left(1+\frac{\boldsymbol{\Delta}^*_{\mathbf{k}}\cdot\boldsymbol{\tau}}{|\boldsymbol{\Delta}_{\mathbf{k}}|}\right)n_F(E_{\mathbf{k},+})+\frac{1}{2}\left(1-\frac{\boldsymbol{\Delta}^*_{\mathbf{k}}\cdot\boldsymbol{\tau}}{|\boldsymbol{\Delta}_{\mathbf{k}}|}\right)n_F(E_{\mathbf{k},-})\right]\delta_{s,s'},\ \text{where}\\
\boldsymbol{\Delta}_{\mathbf{k}} &\equiv (|\Delta_{IVC}(\mathbf{k})|\cos(\phi_{\mathbf{k}}), |\Delta_{IVC}(\mathbf{k})|\sin(\phi_{\mathbf{k}}), \xi_a(\mathbf{k}))\,,\ \text{and}\ E_{\mathbf{k},\pm} = \xi_s(\mathbf{k})\pm|\boldsymbol{\Delta}_{\mathbf{k}}|
\end{aligned} \tag{50}$$

In this case, the first term in Eq. (49) vanishes, and we find:

$$\langle H_{\mathrm{Hund's}}\rangle_{\mathrm{CDW\ IVC}} = \frac{6}{A}\sum_{\mathbf{q},\mathbf{k}} J_H(\mathbf{q})|\lambda^{+-}_{\mathbf{q}}(\mathbf{k})|^2 P_{++}(\mathbf{k})P_{--}(\mathbf{k}+\mathbf{q}) \tag{51}$$

We note that there is an energy penalty for the CDW IVC for local ferromagnetic Hund's ($J_H > 0$) which is proportional to the overlap of Fermi surfaces, at least in the limit where J_H is local, i.e, nearly independent of $\mathbf{q}$. This

is expected, as a local repulsive interaction that gives rise to ferromagnetic Hund's also penalizes accumulation of excess charge density. The converse is true for antiferromagnetic Hund's $J_H < 0$, which arises from a local attractive interaction and favors accumulation of excess charge density.

(ii) Spin-triplet SDW IVC: For this state, the U(2) matrix $U_{ss'} = (\hat{\mathbf{n}} \cdot \mathbf{s})_{ss'}$, upto an overall phase. Using this, the covariance matrix takes the form ($s^0_{s,s'} = \delta_{s,s'}$):

$$P^{s,s'}_{\tau,\tau'} = \frac{1}{2}\left(1 + \frac{[\mathbf{\Delta_k}]^*_{ss'} \cdot \boldsymbol{\tau}}{|\mathbf{\Delta_k}|}\right) n_F(E_{\mathbf{k},+}) + \frac{1}{2}\left(1 - \frac{[\mathbf{\Delta_k}]^*_{ss'} \cdot \boldsymbol{\tau}}{|\mathbf{\Delta_k}|}\right) n_F(E_{\mathbf{k},-}) \text{ where}$$
$$[\mathbf{\Delta_k}]_{ss'} \equiv \big((\hat{\mathbf{n}} \cdot \mathbf{s})|\Delta_{IVC}(\mathbf{k})|\cos(\phi_\mathbf{k}), (\hat{\mathbf{n}} \cdot \mathbf{s})|\Delta_{IVC}(\mathbf{k})|\sin(\phi_\mathbf{k}), s^0\xi_a(\mathbf{k})\big)_{ss'} \tag{52}$$

In this case, both terms in Eq. (49) contribute, and we find:

$$\langle H_{\text{Hund's}}\rangle_{\text{SDW IVC}} = -\frac{4J_H(\mathbf{0})}{A}\left|\sum_\mathbf{k} \lambda^{+-}_{\mathbf{q}=\mathbf{0}}(\mathbf{k})P_{+-}(\mathbf{k})\right|^2 + \frac{6}{A}\sum_{\mathbf{q},\mathbf{k}} J_H(\mathbf{q})|\lambda^{+-}_\mathbf{q}(\mathbf{k})|^2 P_{++}(\mathbf{k})P_{--}(\mathbf{k}+\mathbf{q}) \tag{53}$$

Since the mean-field band structures are identical for both CDW and SDW IVC, we see that the first (Hartree) term gives a contribution which is local in real space, and does not depend on the overlap of Fermi surfaces, while the second (Fock) term will depend on such an overlap. Accordingly, we see that a ferromagnetic Hund's will strongly favor a SDW IVC, while AF Hund's will disfavor it — consistent with our previous arguments. In fact, this can be directly seen by re-writing $H_{\text{Hund's}}$ (for short range Hund's) in terms of the triplet IVC order parameter $\mathbf{n}^{\text{IV}}_{\text{T}}(\mathbf{q})$, which is nothing but $\mathbf{s}_{+-}(\mathbf{q})$:

$$H_{\text{Hund's}} = -\frac{J_H}{A}\sum_\mathbf{q} \mathbf{n}^{\text{IV}}_{\text{T}}(\mathbf{q}) \cdot [\mathbf{n}^{\text{IV}}_{\text{T}}(\mathbf{q})]^\dagger, \text{ where } \mathbf{n}^{\text{IV}}_{\text{T}}(\mathbf{q}) \equiv \sum_\mathbf{k} \lambda^{+-}_\mathbf{q}(\mathbf{k})\psi^\dagger_{+,s,\mathbf{k}}(\hat{\mathbf{n}} \cdot \mathbf{s})_{s,s'}\psi_{-,s',\mathbf{k}+\mathbf{q}} = \mathbf{s}_{+-}(\mathbf{q}) \tag{54}$$

(iii) Spin-polarized IVC: For this state which is relevant at lower doping near SC2, the covariance matrix takes the form (taking spin-polarization axes to be $\hat{z}$):

$$P^{s,s'}_{\tau,\tau'}(\mathbf{k}) = \left[\frac{1}{2}\left(1 + \frac{\mathbf{\Delta}^*_\mathbf{k} \cdot \boldsymbol{\tau}}{|\mathbf{\Delta_k}|}\right) n_F(E_{\mathbf{k},+}) + \frac{1}{2}\left(1 - \frac{\mathbf{\Delta}^*_\mathbf{k} \cdot \boldsymbol{\tau}}{|\mathbf{\Delta_k}|}\right) n_F(E_{\mathbf{k},-})\right] \delta_{s,\uparrow}\delta_{s',\uparrow}, \tag{55}$$

For the spin-polarized (SP) IVC as well, both terms in Eq. (49) contribute, and we find:

$$\langle H_{\text{Hund's}}\rangle_{\text{SP IVC}} = -\frac{J_H(\mathbf{0})}{A}\left|\sum_\mathbf{k} \lambda^{+-}_{\mathbf{q}=\mathbf{0}}(\mathbf{k})P_{+-}(\mathbf{k})\right|^2 + \frac{1}{A}\sum_{\mathbf{q},\mathbf{k}} J_H(\mathbf{q})|\lambda^{+-}_\mathbf{q}(\mathbf{k})|^2 P_{++}(\mathbf{k})P_{--}(\mathbf{k}+\mathbf{q}) \tag{56}$$

We note that a ferromagnetic Hund's term prefers spin-polarization over a singlet IVC, but it prefers SDW IVC over spin-polarized ferromagnetic IVC. Intuitively this happens because the spin-polarized IVC is still a CDW in one-spin species, so although some exchange energy is gained from spin-polarization it is not enough to offset the energy penalty from non-uniform distribution of charge density from a local repulsion that gives rise to ferromagnetic Hund's coupling.

Having discussed in detail the miscroscopically derived Hund's term, we now consider the other symmetry-allowed Hund's coupling, as detailed in Eq. (46), i.e, $\tilde{H}_{\text{Hund's}} = -\frac{\tilde{J}_H}{N}\sum_\mathbf{q} \mathbf{s}_+(\mathbf{q}) \cdot \mathbf{s}_-(-\mathbf{q})$. Note that the above Hamiltonian is Hermitian only if $\tilde{J}_H(\mathbf{q}) = \tilde{J}_H(-\mathbf{q})$, which we will implicitly assume in what follows. The expectation value of this term in a mean-field state is given by:

$$\begin{aligned}
\langle \tilde{H}_{\text{Hund's}}\rangle &= -\frac{1}{A}\sum_{\mathbf{q},\mathbf{k},\mathbf{k}'} \tilde{J}_H(\mathbf{q})\lambda^{++}_\mathbf{q}(\mathbf{k})\lambda^{--}_{-\mathbf{q}}(\mathbf{k}')s^i_{\alpha\beta}s^i_{\mu\nu}\langle\psi^\dagger_{+,\alpha,\mathbf{k}}\psi_{+,\beta,\mathbf{k}+\mathbf{q}}\psi^\dagger_{-,\mu,\mathbf{k}'}\psi_{-,\nu,\mathbf{k}'-\mathbf{q}}\rangle_{\text{IVC}} \\
&= -\frac{\tilde{J}_H(\mathbf{0})}{A}\sum_{\mathbf{k},\mathbf{k}'} \lambda^{++}_{\mathbf{q}=\mathbf{0}}(\mathbf{k})\lambda^{--}_{\mathbf{q}=\mathbf{0}}(\mathbf{k}')(\text{Tr}\big[P_{++}(\mathbf{k})(s^i)^T\big])(\text{Tr}\big[P_{--}(\mathbf{k}')(s^i)^T\big]) \\
&\quad + \frac{1}{N}\sum_{\mathbf{q},\mathbf{k}} \tilde{J}_H(\mathbf{q})\lambda^{++}_\mathbf{q}(\mathbf{k})\lambda^{--}_{-\mathbf{q}}(\mathbf{k}+\mathbf{q})\,\text{Tr}\big[(s^i)^T M (s^i)^T M\big] \\
&= -\frac{\tilde{J}_H(\mathbf{0})}{A}\left(\sum_\mathbf{k}\text{Tr}\big[P_{++}(\mathbf{k})(s^i)^T\big]\right)\left(\sum_{\mathbf{k}'}\text{Tr}\big[P_{--}(\mathbf{k}')(s^i)^T\big]\right) \\
&\quad + \frac{1}{A}\sum_{\mathbf{q},\mathbf{k}} \tilde{J}_H(\mathbf{q})\lambda^{++}_\mathbf{q}(\mathbf{k})[\lambda^{--}_\mathbf{q}(\mathbf{k})]^*\,\text{Tr}\big[(s^i)^T P_{+-}(\mathbf{k})(s^i)^T P_{-+}(\mathbf{k}+\mathbf{q})\big]
\end{aligned} \tag{57}$$

where $P^{s,s'}_{\tau,\tau'}$ is the projector onto the HF mean-field IVC ground state, the trace is over spin degrees of freedom, and we have used $\lambda^{\tau\tau}_{\mathbf{q}=0}(\mathbf{k}) = 1$ by virtue of normalization of Bloch-wavefunctions. Note that for unitary IVC, the first term vanishes as the valley-diagonal projectors are proportional to $\delta_{s,s'}$. Further, the second term always features non-trivial winding, as discussed earlier in the context of IVC energetics. In particular, we note that $P_{+-}(\mathbf{k}) \sim e^{-i\phi_{\mathbf{k}}}$, and $\lambda^{++}_{\mathbf{q}}(\mathbf{k})[\lambda^{--}_{\mathbf{q}}(\mathbf{k})]^* \sim e^{-i\mathbf{q}\cdot(\boldsymbol{A}_+ - \boldsymbol{A}_-)}$ where $\boldsymbol{A}_\pm(\mathbf{k})$ denote the Berry-connection in the $\tau = \pm$ valleys (see previous discussion on IVC energetics for a detailed description). So the sign of this term is not uniform and its effect will be quite small if $\tilde{J}_H(\mathbf{q})$ is local, i.e, approximately independent of $\mathbf{q}$. Therefore, even if it is present, we generally expect the effect of this term to be quite small for unitary IVCs. More explicitly, we have the following contributions for the three kinds of IVC states.

(i) Spin-singlet CDW IVC is weakly favored by antiferromagnetic coupling ($\tilde{J}_H < 0$), as:

$$\langle \tilde{H}_{\text{Hund's}} \rangle_{\text{CDW IVC}} = \frac{6}{A} \sum_{\mathbf{q},\mathbf{k}} \tilde{J}_H(\mathbf{q}) \lambda^{++}_{\mathbf{q}}(\mathbf{k}) [\lambda^{--}_{\mathbf{q}}(\mathbf{k})]^* P_{+-}(\mathbf{k}) P_{-+}(\mathbf{k}+\mathbf{q}) \tag{58}$$

(ii) Spin-triplet SDW IVC is weakly favored by ferromagnetic coupling ($\tilde{J}_H > 0$), as:

$$\langle \tilde{H}_{\text{Hund's}} \rangle_{\text{SDW IVC}} = -\frac{2}{A} \sum_{\mathbf{q},\mathbf{k}} \tilde{J}_H(\mathbf{q}) \lambda^{++}_{\mathbf{q}}(\mathbf{k}) [\lambda^{--}_{\mathbf{q}}(\mathbf{k})]^* P_{+-}(\mathbf{k}) P_{-+}(\mathbf{k}+\mathbf{q}) \tag{59}$$

(iii) Spin-polarized IVC: For such a state, the expectation value of $H_{\text{Hund's}}$ includes both a *Hartree* and *Fock* contribution, unlike the previous two cases where the *Hartree* contribution was zero due to lack of net spin-polarization. Taking the spin-quantization axis to be $\hat{\mathbf{n}} = \hat{z}$, we have (using $\lambda^{\tau\tau}_{\mathbf{q}=0}(\mathbf{k}) = 1$):

$$\langle \tilde{H}_{\text{Hund's}} \rangle_{\text{SP IVC}} = -\frac{\tilde{J}_H(\mathbf{0})}{A} \left(\sum_{\mathbf{k}} P_{++}(\mathbf{k}) \right) \left(\sum_{\mathbf{k}'} P_{--}(\mathbf{k}') \right) + \frac{1}{A} \sum_{\mathbf{q},\mathbf{k}} \tilde{J}_H(\mathbf{q}) \lambda^{++}_{\mathbf{q}}(\mathbf{k}) [\lambda^{--}_{\mathbf{q}}(\mathbf{k})]^* P_{+-}(\mathbf{k}) P_{-+}(\mathbf{k}+\mathbf{q}) \tag{60}$$

The first term involves a sum over $\mathbf{k}$, and is therefore completely local. In fact, since the total number of dopants is given by $\sum_{\mathbf{k},\tau} P_{\tau\tau}(\mathbf{k}) = N_h$, if we assume that only one band is filled then this term just gives the net alignment energy of all the spins $-J_H N_h^2/4A$. The second term can be thought of as a *Fock* contribution, which depends on the overlap of Fermi surfaces of the mean-field bands when displaced by $\mathbf{q}$, and the product of the form factors which decay with $\mathbf{q}$. Therefore, it decays fast a function of $\mathbf{q}$, and also contains non-trivial winding which further decreases the overall magnitude of this contribution. Thus, the *Hartree* contribution dominates, and aids spin-polarization for FM Hund's $\tilde{J}_H > 0$ (opposes it or penalizes a spin-polarized IVC for AFM Hund's $\tilde{J}_H < 0$).

To summarize, the microscopically derived Hund's coupling J_H prefers the SDW IVC when it originates from spatially local repulsive interaction and is ferromagnetic, and it prefers the CDW IVC when it originates from local attractive interactions and is antiferromagnetic. This follows from the observation that local attractive interactions favor a CDW, while local repulsive interactions prefer equal charge density on all sites. The spin-polarized IVC state (which is also a CDW) is only weakly favored by a ferromagnetic Hund's term, as the exchange energy gained by aligning spins competes with the energy penalty from excess charge density accumulation. In contrast, even though a Hund's coupling with $\tilde{J}_H$ is symmetry-allowed, it does not directly arise within our microscopic calculation, and is expected to be quite small. This form of Hund's term (which is, for example, discussed in Ref. 2) has a weak effect on the IVC states, and prefers spin-polarization instead when it is ferromagnetic. When antiferromagnetic, it prefers the spin-singlet CDW IVC, but only weakly due to the winding of form-factors.

B. Isospin polarized states without IVC

Since the experiment [2, 15] finds spin-polarization at smaller hole-doping, it is natural to ask if our proposed Hund's term can lead to spin-polarization while valley remains a good quantum number. Here we show that ferromagnetic J_H can indeed favor such spin-polarized states over a spin-valley locked state (which has oppositely aligned spins for the valleys), and over a valley-polarized phase. For this purpose, we will evaluate $\langle H_{\text{Hund's}} \rangle$ for (a) a spin-polarized ferromagnetic state, and (b) a spin-valley locked state, with spins pointing in opposite directions in the two valleys.

$$\begin{aligned} \langle H_{\text{Hund's}} \rangle_{\text{SP/SVL}} &= -\frac{1}{A} \sum_{\mathbf{q},\mathbf{k},\mathbf{k}'} J_H(\mathbf{q}) \lambda^{+-}_{\mathbf{q}}(\mathbf{k}) \lambda^{-+}_{-\mathbf{q}}(\mathbf{k}') s^i_{\alpha\beta} s^i_{\mu\nu} \left(P^{\alpha\beta}_{+-}(\mathbf{k}) P^{\mu\nu}_{-+}(\mathbf{k}') \delta_{\mathbf{q},\mathbf{0}} - P^{\alpha\nu}_{++}(\mathbf{k}) P^{\mu\beta}_{--}(\mathbf{k}') \delta_{\mathbf{k}',\mathbf{k}+\mathbf{q}} \right) \\ &= \frac{1}{A} \sum_{\mathbf{q},\mathbf{k}} J_H(\mathbf{q}) |\lambda^{+-}_{\mathbf{q}}(\mathbf{k})|^2 \,\mathrm{Tr}\big[P_{++}(\mathbf{k}) (s^i)^T P_{--}(\mathbf{k}+\mathbf{q}) (s^i)^T \big] \end{aligned} \tag{61}$$

where we have noted that in both cases, $P^{s,s'}_{\tau,\tau'} \propto \delta_{s,s'}\delta_{\tau,\tau'}$ so the Hartree term does not contribute.

(a) Spin-polarized ferromagnet: The covariance matrix is given by $P^{s,s'}_{\tau,\tau'}(\mathbf{k}) = P_{\tau,\tau'}(\mathbf{k})\left(\frac{1+\hat{\mathbf{n}}\cdot\mathbf{s}}{2}\right)_{s,s'}$. Choosing $\hat{\mathbf{n}} = \hat{z}$ for simplicity (although our answer does not depend on this choice), we find that:

$$\langle H_{\text{Hund's}}\rangle_{\text{SP}} = \frac{1}{A}\sum_{\mathbf{q},\mathbf{k}} \tilde{J}_H(\mathbf{q})|\lambda^{+-}_{\mathbf{q}}(\mathbf{k})|^2 P_{++}(\mathbf{k})P_{--}(\mathbf{k}+\mathbf{q}) \tag{62}$$

(b) Spin-valley locked state: This state has spins pointing in opposite directions $\pm\hat{\mathbf{n}}$ in the two valleys, with a covariance matrix given by $P^{s,s'}_{\tau,\tau'}(\mathbf{k}) = P_{\tau,\tau'}(\mathbf{k})\left(\frac{1+\tau\hat{\mathbf{n}}\cdot\mathbf{s}}{2}\right)_{s,s'}$. Once again, choosing $\hat{\mathbf{n}} = \hat{z}$ for simplicity, we have:

$$\langle H_{\text{Hund's}}\rangle_{\text{SVL}} = \frac{2}{A}\sum_{\mathbf{q},\mathbf{k}} \tilde{J}_H(\mathbf{q})|\lambda^{+-}_{\mathbf{q}}(\mathbf{k})|^2 P_{++}(\mathbf{k})P_{--}(\mathbf{k}+\mathbf{q}) \tag{63}$$

Thus, this Hund's term has a larger penalty for the spin-valley locked state compared to the spin-polarized state, and thus favors the spin-polarized state. Note that this is exactly the kind of behavior that would also be a consequence of the other kind of Hund's term $\tilde{H}_{\text{Hund's}}$ in Eq. (46), which will directly favor the spin-polarized phase over the spin-valley locked phase at the Hartree level. The surprising feature is that even $H_{\text{Hund's}}$ in Eq. (5) in the main text chooses the same term, and microscopically the origin of this lies at noting that a locally repulsive interaction that gives rise to ferromagnetic J_H will naturally favor a spatially anti-symmetric wavefunction to minimize local replusion, leading to spin polarization.

Since $\langle H_{\text{Hund's}}\rangle = 0$ for a valley-polarized state, one might be tempted to conclude that it favors valley-polarization over spin-polarization. However, this conclusion is incorrect. The reason is that the $\text{SU}(2)_+\times\text{SU}(2)_-$ symmetric interaction terms that we discarded following the application of the Fierz identity in Eq. (42) play an important role in determining the energy difference between spin and valley polarized states, as these terms break the putative SU(4) symmetry that allows rotating between spin and valley degrees of freedom. Therefore, we should start directly with Eq. (41) to see that this is not the case, and spin-polarization is favored over valley-polarization, as we do explicitly next. For simplicity, we assume $U(\mathbf{r}-\mathbf{r}')$ is short-range, and does not depend on sublattice index a, i.e, $U_{ab}(\mathbf{q}+2\tau\mathbf{K}) = U$. Then, we have:

$$H_{\text{inter-valley}} = \frac{U}{2A}\sum_{\mathbf{k},\mathbf{k}',\mathbf{q},\tau} \lambda^{-\tau,\tau}_{\mathbf{q}}(\mathbf{k})\lambda^{\tau,-\tau}_{-\mathbf{q}}(\mathbf{k}') : \psi^\dagger_{-\tau,s,\mathbf{k}}\psi_{\tau,s,\mathbf{k}+\mathbf{q}}\psi^\dagger_{\tau,s',\mathbf{k}'}\psi_{-\tau,s',\mathbf{k}'-\mathbf{q}} : \tag{64}$$

For any-valley and spin-diagonal ansatz, i.e, $P^{s,s'}_{\tau,\tau'}(\mathbf{k}) \neq 0$ only if $\tau = \tau'$ and $s = s'$ we note that only the Fock term contributes. Therefore:

$$\langle H_{\text{inter-valley}}\rangle = -\frac{U}{2A}\sum_{\mathbf{k},\mathbf{q}} |\lambda^{\tau,-\tau}_{\mathbf{q}}(\mathbf{k})|^2 \,\text{Tr}\left[P_{\tau,\tau}(\mathbf{k})P_{-\tau,-\tau}(\mathbf{k}+\mathbf{q})\right] \tag{65}$$

Using this, we see that:

$$\langle H_{\text{inter-valley}}\rangle = \begin{cases} -\frac{U}{2A}\sum_{\mathbf{k},\mathbf{q},\tau} |\lambda^{\tau,-\tau}_{\mathbf{q}}(\mathbf{k})|^2 P_{\tau,\tau}(\mathbf{k})P_{-\tau,-\tau}(\mathbf{k}+\mathbf{q}), & \text{spin-polarized} \\ 0, & \text{spin-valley locked} \\ 0, & \text{valley-polarized} \end{cases} \tag{66}$$

Thus, a locally repulsive interaction ($U > 0$) prefers the spin-polarized (ferromagnetic) state over the spin-valley locked state (spins oppositely aligned in the two valleys) and valley-polarized state. Note, however, that the latter two are not affected by the inter-valley coupling to lowest order in perturbation theory — a conclusion that relies on perturbatively studying the full inter-valley scattering term (and not just the sign of inter-valley Hund's coupling).

Supplementary Note 6. SUPERCONDUCTIVITY

A. Symmetries of pairing correlations

In this section, we elaborate on the symmetries of the pairing correlation function. The action of the symmetry operators on the c fermions are given in Eq. (5), the same action carries over to the ψ fermions since these are related

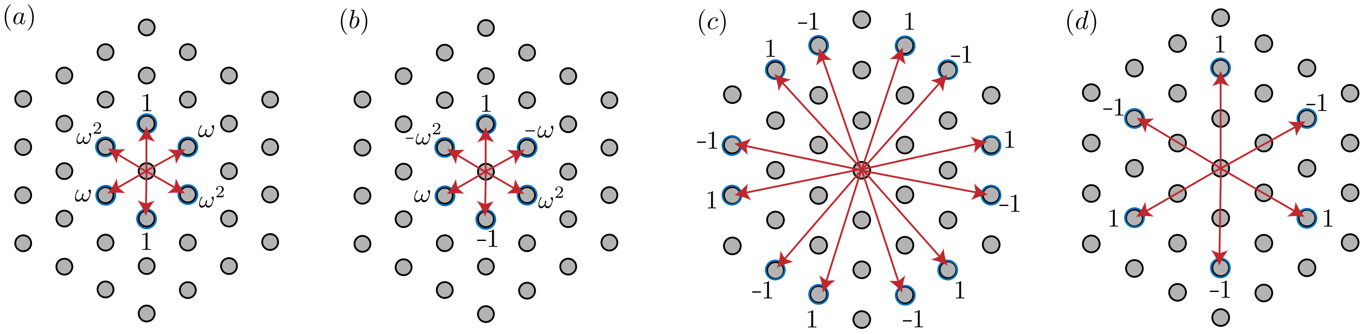

Supplementary Figure 6. Recipe for extension of pair-correlation functions to the full BZ for (a) chiral spin-singlet, $d+id$, (b) chiral spin-triplet, $p+ip$, (c) nodal spin-singlet, i-wave, and (d) nodal spin-triplet, f-wave. The momentum-dependent part in each case is given by $\Delta(\mathbf{q}) \sim \sum_i c_i e^{i\mathbf{q}\cdot\mathbf{a}_i}$, where the required lattice translation vectors $\mathbf{a}_i$ corresponding to the real space triangular lattice formed by A_1/B_3 are shown by red arrows, and the corresponding weights c_i (when non-zero) are noted beside each site.

by a change of basis. In the most general scenario, the pair-correlation function 4×4 matrix in spin and valley space: $F^{\tau,\tau'}_{s,s'}(\mathbf{k}) = \langle \psi_{\tau,s,-\mathbf{k}}\psi_{\tau',s',\mathbf{k}}\rangle$. Anticommutation of the fermion field operators implies that:

$$F^{\tau,\tau'}_{s,s'}(\mathbf{k}) = -F^{\tau',\tau}_{s',s}(-\mathbf{k}) \tag{67}$$

We focus on inter-valley pairing so that $\tau' = -\tau$, so that we can write $F^{\tau,\tau'}_{s,s'}(\mathbf{k}) = \tau^\alpha F_{s,s'}(\mathbf{k})$, with $\alpha = x$ or y, and $F_{s,s'}(\mathbf{k}) = \langle \psi_{-,s,-\mathbf{k}}\psi_{+,s',\mathbf{k}}\rangle$ is 2×2 matrix in spin-space.

We first classify $F_{s,s'}(\mathbf{k})$ by the action of global spin-rotation symmetry $\mathrm{SU}(2)_s$. Spin-singlet pair-correlations are invariant under $\mathrm{SU}(2)_s$ and can be written as $F_{s,s'}(\mathbf{k}) = is^y f_\mathbf{k}$, while (unitary) spin-triplet correlations transform as a vector under $\mathrm{SU}(2)_s$ and can be written as $F_{s,s'}(\mathbf{k}) = is^y(\hat{\mathbf{d}}\cdot\mathbf{s})f_\mathbf{k}$, where $\hat{\mathbf{d}}$ is a real unit-vector. However, we note that $f_\mathbf{k}$ can be either odd or even under $\mathbf{k}\to-\mathbf{k}$ for both singlets and triplets, depending on the choice of τ^y or τ^x for the inter-valley pairing, such that $F^{\tau,\tau'}_{s,s'}(\mathbf{k})$ is appropriately antisymmetric (see Eq. (67)) .

Next we discuss spatial symmetries. Since we consider inter-valley pairing, $f_\mathbf{k}$ is invariant under translation symmetry. We may also classify by C_3 rotations about the K/K' points, since $\mathbf{k}$ is measured relative to these points in the BZ. Since C_3 is a symmetry about K/K' points [4], we have $f_\mathbf{k}\to f_{C_3\mathbf{k}} = e^{2\pi i L_z/3}f_\mathbf{k}$, where $L_z = 0,1,2$ are distinct. Note that $L_z = 3$ transforms trivially under C_3, and cannot be directly used to distinguish order parameters of the form $f_\mathbf{k} = \mathrm{Im}[(k_x+ik_y)^3]$ from $f_\mathbf{k}$ independent of $\mathbf{k}$ ($L_z = 0$). However, there is an additional mirror symmetry M_x, as we discussed previously (see Eq. (5)). Consider the spin-singlet nodal order parameter $F(\mathbf{k}) = \tau^y s^y f_\mathbf{k}$, with $f_\mathbf{k} = \mathrm{Im}[(k_x+ik_y)^3] = k_y(3k_x^2-k_y^2)$. Then, under $M_x\tilde{\mathcal{T}}$, where $\tilde{\mathcal{T}}$ is spinless time-reversal, $f_\mathbf{k}\to f^*_{M_y(\mathbf{k})} = -f_\mathbf{k}$, where $M_y(\mathbf{k}) = (k_x,-k_y)$. So the spin-singlet nodal superconductor is odd under $M_x\tilde{\mathcal{T}}$, with nodes at $k_y = 0$ and C_3-related points in the Brillouin Zone. In contrast, the fully-gapped spin-singlet s-wave with $F(\mathbf{k}) = \tau^x s^y$ is even under $M_x\tilde{\mathcal{T}}$. Thus, the nodal superconductor is distinguished from the gapped s-wave superconductor by $M_x\tilde{\mathcal{T}}$. Finally, we comment that $\mathrm{Im}[(k_x+ik_y)^3]$ and $\mathrm{Re}[(k_x+ik_y)^3]$ transform as distinct irreps of the symmetry group, and therefore are not energetically degenerate, as can be seen from the fact that $\mathrm{Re}[(k_x+ik_y)^3]$ is even under $M_x\tilde{\mathcal{T}}$. This is unlike $\mathrm{Re}(k_x+ik_y)$ and $\mathrm{Im}(k_x+ik_y)$ which belong to a single two-dimensional irrep, and are degenerate.

It is also instructive to consider the extensions of the pairing wave-functions from small momenta patches around the K/K' points, to the entire BZ. Fermionic anticommutation relations constrain that for a spin-singlet (spin-triplet) superconductor, such a pair-correlation function is necessarily even (odd) in the momentum $\mathbf{q}$ measured relative to the Γ-point in the BZ, i.e, have definite transformation under $C_2:\mathbf{q}\to-\mathbf{q}$. To construct the simplest extensions (with least number of nodes) of the pair-correlations that are compatible with their behavior around K,K' points and periodic in the BZ (i.e, obey lattice translation symmetry), we consider functions of the form $\Delta(\mathbf{q}) \sim \sum_i c_i e^{i\mathbf{q}\cdot\mathbf{a}_i}$, where c_i are complex weights that are chosen to be consistent with the behavior under C_3 (about Γ) and M_x, and $\mathbf{a}_i$ form the minimal set of lattice translation vectors (on the triangular lattice formed by A_1/B_3) that allow us to impose the required odd/even behavior under M_x and C_2. These choices are shown pictorially in Supplementary Figure 6. In particular, note that simplest extension compatible with the behavior of the chiral gapped superconductor ($L_z = 1$ about K) is $d_{x^2-y^2}+id_{xy}$ for a spin-singlet, and p_x+ip_y for a spin-triplet. For the nodal superconductor, the structure is more complicated. For the spin-triplet, it is f-wave (angular momentum 3 about the Γ point), while for the spin-singlet, it is i-wave (angular momentum 6 about the Γ point).

B. Derivation of gap equations

In this section, we derive superconducting gap equations, which we solve numerically, in different scenarios. To this end, we will project all interactions onto the valence bands, which is a reasonable approximation as long as the typical interaction energy scales are less relative to the band-gap. To check this, note that near the K (or K′) point, the bands are quite flat and the displacement-field induced gap between the valence band and the conduction band is around 64 meV at $u = 30$ meV. The typical interaction scale, for unscreened Coulomb interaction, is given by $E_C = e^2/4\pi\epsilon\epsilon_0\langle r\rangle$, where $\langle r\rangle \approx 1/\sqrt{n_h}$ is the average separation between the charge carriers. For the experimentally relevant carrier-density $n_h \sim 10^{12}$ cm^{-2} and hBN dielectric constant $\epsilon = 4.4$, we find that $E_C \approx 33$ meV, which is smaller than the band-gap, albeit not by an order of magnitude. Dual-gate screening reduces this estimate to $E_C \approx 28$ meV, and screening due to itinerant hole-carriers in the sample itself will further decrease E_C. In addition, the matrix elements coming from imperfect wavefunction overlaps between the valence and conduction bands would act to suppress interband scattering. Therefore, it is reasonable to project the interaction onto the valence band to analyze fluctuation-mediated superconductivity.

We first consider IVC fluctuations in the SU(2)$_+\times$SU(2)$_-$ symmetric limit where spin-singlet and triplet superconductors are degenerate, and then turn to their splitting due to relative amplification of CDW vs SDW fluctuations due to Hund's coupling. Simultaneously, we also consider spin-polarized IVC fluctuations which may be relevant for SC2. Finally, we will also consider the effect of Coulomb interactions on pairing at a mean-field level.

In the SU(2)$_+\times$SU(2)$_-$ symmetric limit, a phenomenological Hamiltonian for IVC fluctuations is given by:

$$H^{\text{eff}}_{\text{IVC}} = -\frac{1}{A}\sum_{\mathbf{q}} g_{\mathbf{q}}\ \text{Tr}\left[n^{\text{IV}}(\mathbf{q})[n^{\text{IV}}(\mathbf{q})]^\dagger\right],,\ \text{where } n^{\text{IV}}_{s,s'}(\mathbf{q}) = \sum_{\mathbf{k}} \lambda^{+-}_{\mathbf{q}}(\mathbf{k})\psi^\dagger_{+,s,\mathbf{k}}\psi_{-,s'\mathbf{k}+\mathbf{q}},\ \text{and } g_{\mathbf{q}} = \frac{g}{\mathbf{q}^2+\xi^{-2}_{IVC}} \tag{68}$$

We decouple this interaction in the BCS channel:

$$H^{\text{eff}}_{\text{IVC}} \xrightarrow{\mathbf{q}=-(\mathbf{k}+\mathbf{k}')} \frac{1}{A}\sum_{\mathbf{k},\mathbf{k}'} V_{\mathbf{k},\mathbf{k}'}\psi^\dagger_{+,s,\mathbf{k}}\psi^\dagger_{-,s',-\mathbf{k}}\psi_{-,s',-\mathbf{k}'}\psi_{+,s,\mathbf{k}'},\ \text{with } V_{\mathbf{k},\mathbf{k}'} = g_{\mathbf{q}=-\mathbf{k}-\mathbf{k}'}|\lambda^{+-}_{\mathbf{q}=-(\mathbf{k}+\mathbf{k}')}(\mathbf{k})|^2 \tag{69}$$

Note that the effective interaction $V_{\mathbf{k},\mathbf{k}'}$ in the valence band projected basis is symmetric under $\mathbf{k}\to\mathbf{k}'$, and it is repulsive. Further, it is maximum at $\mathbf{k}' = -\mathbf{k}$, stemming for the fact that both $g_{\mathbf{q}}$ is maximized at $\mathbf{q} = 0$, and and $|\lambda^{+-}_{\mathbf{q}}(\mathbf{k})|$ is relatively featureless at small $|\mathbf{q}$ and $|\mathbf{k}|$ due to substantial sublattice polarization. We define the mean-field superconducting gap matrix (in spin-space):

$$\Delta_{ss'}(\mathbf{k}) = \frac{1}{A}\sum_{\mathbf{k}} V_{\mathbf{k},\mathbf{k}'}\langle\psi_{-,s,-\mathbf{k}'}\psi_{+,s',\mathbf{k}}\rangle = \frac{1}{A}\sum_{\mathbf{k}} V_{\mathbf{k},\mathbf{k}'}F_{ss'}(\mathbf{k}') \tag{70}$$

This implies that:

$$\Delta^*_{ss'}(\mathbf{k}) = \frac{1}{A}\sum_{\mathbf{k}} V_{\mathbf{k},\mathbf{k}'}\langle\psi^\dagger_{+,s',\mathbf{k}}\psi^\dagger_{-,s,-\mathbf{k}'}\rangle \tag{71}$$

Now, we perform a mean-field decoupling of $H^{\text{eff}}_{\text{IVC}}$ as follows:

$$H_{\text{IVC}} \xrightarrow{\text{mean field}} \sum_{\mathbf{k},s,s'} \Delta^*_{s's}(\mathbf{k})\psi_{-,s',-\mathbf{k}'}\psi_{+,s,\mathbf{k}} + \Delta_{s's}(\mathbf{k})\psi^\dagger_{+,s,\mathbf{k}}\psi^\dagger_{-,s',-\mathbf{k}'} - A\sum_{\mathbf{k},\mathbf{k}',s,s'} \Delta^*_{s's}(\mathbf{k})V^{-1}_{\mathbf{k},\mathbf{k}'}\Delta_{s's}(\mathbf{k}') \tag{72}$$

where by V^{-1} we have denoted the inverse of the matrix $V_{\mathbf{k},\mathbf{k}'}$ in momentum space. Therefore, the total mean-field Hamiltonian can be written in the Nambu (particle-hole) space as:

$$\begin{aligned} H_{MF} &= H_{\text{BdG}} - A\sum_{\mathbf{k},\mathbf{k}',s,s'} \Delta^*_{s's}(\mathbf{k})V^{-1}_{\mathbf{k},\mathbf{k}'}\Delta_{s's}(\mathbf{k}'),\ \text{where} \\ H_{\text{BdG}} &= \sum_{\mathbf{k},s,s'} \begin{pmatrix}\psi^\dagger_{+,s,\mathbf{k}} & \psi_{-,s',-\mathbf{k}}\end{pmatrix} \begin{pmatrix}\xi_{+,\mathbf{k}} & \Delta_{s's}(\mathbf{k}) \\ \Delta^*_{s's}(\mathbf{k}) & -\xi_{-,-\mathbf{k}}\end{pmatrix} \begin{pmatrix}\psi_{+,s,\mathbf{k}} \\ \psi^\dagger_{-,s',-\mathbf{k}}\end{pmatrix} \end{aligned} \tag{73}$$

The free-energy of the system can be written as:

$$\mathcal{F} = -\frac{1}{\beta}\ln(Z) - A\sum_{\mathbf{k},\mathbf{k}',s,s'} \Delta^*_{s's}(\mathbf{k})V^{-1}_{\mathbf{k},\mathbf{k}'}\Delta_{s's}(\mathbf{k}') \tag{74}$$

where Z is the partition function of the system, given in terms of a sum over momenta and fermionic Matsubara frequencies $\omega_n = (2n+1)\pi/\beta$:

$$\frac{1}{\beta}\ln(Z) = \frac{1}{\beta}\sum_{\mathbf{k},i\omega_n,s,s'} \ln\left[(-i\omega_n + \xi_+(\mathbf{k}))(-i\omega_n - \xi_-(-\mathbf{k})) - |\Delta_{s's}(\mathbf{k})|^2\right] \tag{75}$$

Now, we expand the logarithm in a power series in $\Delta_{s's}(\mathbf{k})$, with the aim to derive a gap equation close to T_c where the gap goes to zero.

$$\begin{aligned}\frac{1}{\beta}\ln(Z) &\approx \text{const.} - \sum_{\mathbf{k},s,s'} |\Delta_{s's}(\mathbf{k})|^2 \left(\frac{1}{\beta}\sum_{\omega_n}\frac{1}{(-i\omega_n + \xi_+(\mathbf{k}))(-i\omega_n - \xi_-(-\mathbf{k}))}\right)\\ &= \text{const.} - \sum_{\mathbf{k},s,s'} |\Delta_{s's}(\mathbf{k})|^2 \left(\frac{1-2n_F(\xi_+(\mathbf{k}))}{2\xi_+(\mathbf{k})}\right)\end{aligned} \tag{76}$$

where we have used time-reversal symmetry to set $\xi_-(-\mathbf{k}) = \xi_+(\mathbf{k})$, and $n_F(\xi) = (\exp(\beta\xi)+1)^{-1}$ is the Fermi-function. This leads to the following expression for the free energy:

$$\mathcal{F} = \text{const.} + \sum_{\mathbf{k},s,s'} |\Delta_{s's}(\mathbf{k})|^2 \left(\frac{1-2n_F(\xi_+(\mathbf{k}))}{2\xi_+(\mathbf{k})}\right) - A\sum_{\mathbf{k},\mathbf{k}',s,s'} \Delta^*_{s's}(\mathbf{k}) V^{-1}_{\mathbf{k},\mathbf{k}'}\Delta_{s's}(\mathbf{k}') \tag{77}$$

The gap-equation can be derived setting the variation the free energy with respect to the gap function to be zero.

$$\begin{aligned}\frac{\partial\mathcal{F}}{\partial(\Delta^*_{s's}(\mathbf{k}))} = 0 &\implies \Delta_{s's}(\mathbf{k})\left(\frac{1-2n_F(\xi_+(\mathbf{k}))}{2\xi_+(\mathbf{k})}\right) - A\sum_{\mathbf{k}'} V^{-1}_{\mathbf{k},\mathbf{k}'}\Delta_{s's}(\mathbf{k}') = 0\\ &\implies \Delta_{s's}(\mathbf{k}) = -\frac{1}{A}\sum_{\mathbf{k}'} V_{\mathbf{k},\mathbf{k}'}\left(\frac{\tanh(\beta\xi_+(\mathbf{k}')/2)}{2\xi_+(\mathbf{k}')}\right)\Delta_{s's}(\mathbf{k}')\end{aligned} \tag{78}$$

For spin-singlet superconductors, we can write $\Delta(\mathbf{k}) = is^y f_{\mathbf{k}}$, where $f_{\mathbf{k}}$ is a scalar. For spin-triplet superconductors, we can write $\Delta(\mathbf{k}) = (\mathbf{d}\cdot\mathbf{s})is^y f_{\mathbf{k}}$, where $\mathbf{d}$ is a unit-vector. In both cases, we find the following equation for the 'orbital' part of the pair-wavefunction $f_{\mathbf{k}}$:

$$f_{\mathbf{k}} = -\frac{1}{A}\sum_{\mathbf{k}'} V_{\mathbf{k},\mathbf{k}'}\left(\frac{\tanh(\beta\xi_+(\mathbf{k}')/2)}{2\xi_+(\mathbf{k}')}\right) f_{\mathbf{k}'} \tag{79}$$

Since $V_{\mathbf{k},\mathbf{k}'}$ is maximized near $\mathbf{k}+\mathbf{k}'=0$, we may focus on this parameter regime near the Fermi surface. Since V is repulsive, this means that the gap equation can be satisfied when $f_{\mathbf{k}'=-\mathbf{k}} = -f_{\mathbf{k}}$, which indicates unconventional pairing. The actual channel is determined by the IVC correlation length ξ_{IVC}, for small ξ_{IVC} it is a nodal and non-chiral, while for large ξ_{IVC} it is gapped and chiral as explained in the main text.

As discussed in the main text, introducing a Hund's coupling will amplify either singlet and or triplet IVC fluctuations, and one important consequence of this is the splitting of the degeneracy between singlet and triplet superconductors. Therefore, we start with Eq. (10) in the main text (reproduced below for convenience), where the effective IVC interaction Hamiltonian $H^{\text{eff}}_{\text{IVC}}$ was decomposed into singlet (CDW) and triplet (SDW) IVC fluctuations with distinct susceptibilities $g^{\text{S}}_{\mathbf{q}}$ and $g^{\text{T}}_{\mathbf{q}}$ respectively:

$$H^{\text{eff}}_{\text{IVC}} = -\frac{1}{2A}\sum_{\mathbf{q}} g^{\text{S}}_{\mathbf{q}}\,\text{Tr}\left[n^{\text{IV}}_{\text{S}}(\mathbf{q})[n^{\text{IV}}_{\text{S}}(\mathbf{q})]^\dagger\right] - \frac{1}{2A}\sum_{\mathbf{q}} g^{\text{T}}_{\mathbf{q}}\,\text{Tr}\left[n^{\text{IV}}_{\text{T}}(\mathbf{q})[n^{\text{IV}}_{\text{T}}(\mathbf{q})]^\dagger\right] \tag{80}$$

Now, we derive the linearized gap equation for each of the two terms in Eq. (80), and then combine these to get the gap equation when both are present. We follow the same procedure as described for the fully symmetric scenario, so we only outline the basic steps here. For the singlet IVC channel, the BCS decoupling takes the following form:

$$\begin{aligned}-\frac{1}{2A}\sum_{\mathbf{q}} g^{\text{S}}_{\mathbf{q}}\,\text{Tr}\left[n^{\text{IV}}_{\text{S}}(\mathbf{q})[n^{\text{IV}}_{\text{S}}(\mathbf{q})]^\dagger\right] \xrightarrow{\text{mean field}} &\sum_{\mathbf{k},s,s'} \Delta^*_{s's}(\mathbf{k})\psi_{-,s,-\mathbf{k}'}\psi_{+,s',\mathbf{k}} + \Delta_{ss'}(\mathbf{k})\psi^\dagger_{+,s,\mathbf{k}}\psi^\dagger_{-,s',-\mathbf{k}'}\\ &-A\sum_{\mathbf{k},\mathbf{k}',s,s'} \Delta^*_{ss'}(\mathbf{k})[V^{\text{S}}_{\mathbf{k},\mathbf{k}'}]^{-1}\Delta_{s's}(\mathbf{k}'),\end{aligned}$$

$$\text{where } V^{\text{S}}_{\mathbf{k},\mathbf{k}'} = \frac{1}{2} g^{\text{S}}_{\mathbf{q}=-\mathbf{k}-\mathbf{k}'}|\lambda^{+-}_{\mathbf{q}=-(\mathbf{k}+\mathbf{k}')}(\mathbf{k})|^2 \text{ and } \Delta_{ss'}(\mathbf{k}) = \frac{1}{A}\sum_{\mathbf{k}} V^{\text{S}}_{\mathbf{k},\mathbf{k}'}\langle\psi_{-,s,-\mathbf{k}'}\psi_{+,s',\mathbf{k}}\rangle \tag{81}$$

Therefore, the total mean-field Hamiltonian can be written as:

$$H_{MF} = H_{\rm BdG} - A \sum_{\mathbf{k},\mathbf{k}',s,s'} \Delta^*_{s's}(\mathbf{k})[V^{\rm S}_{\mathbf{k},\mathbf{k}'}]^{-1}\Delta_{ss'}(\mathbf{k}'), \text{ where}$$
$$H_{\rm BdG} = \sum_{\mathbf{k},s,s'} \begin{pmatrix}\psi^\dagger_{+,s,\mathbf{k}} & \psi_{-,s',-\mathbf{k}}\end{pmatrix} \begin{pmatrix}\xi_{+,\mathbf{k}} & \Delta_{ss'}(\mathbf{k}) \\ \Delta^*_{ss'}(\mathbf{k}) & -\xi_-(-\mathbf{k})\end{pmatrix} \begin{pmatrix}\psi_{+,s,\mathbf{k}} \\ \psi^\dagger_{-,s',-\mathbf{k}}\end{pmatrix} \tag{82}$$

Evaluating the free energy and minimizing it leads to:

$$\Delta_{s's}(\mathbf{k}) = -\frac{1}{A}\sum_{\mathbf{k}'} V_{\mathbf{k},\mathbf{k}'}\left(\frac{\tanh(\beta\xi_+(\mathbf{k}')/2)}{2\xi_+(\mathbf{k}')}\right)\Delta_{ss'}(\mathbf{k}') \tag{83}$$

In turn, this implies the following equation for the spatial wave-functions of spin-singlet and spin-triplet superconductors:

$$f_{\mathbf{k}} = \begin{cases} \frac{1}{A}\sum_{\mathbf{k}'} V^{\rm S}_{\mathbf{k},\mathbf{k}'}\left(\frac{\tanh\left(\beta\xi_+(\mathbf{k}')/2\right)}{2\xi_+(\mathbf{k}')}\right) f_{\mathbf{k}'}, \text{ for spin-singlets: } \Delta(\mathbf{k}) = is^y f_{\mathbf{k}} \\ -\frac{1}{A}\sum_{\mathbf{k}'} V^{\rm S}_{\mathbf{k},\mathbf{k}'}\left(\frac{\tanh\left(\beta\xi_+(\mathbf{k}')/2\right)}{2\xi_+(\mathbf{k}')}\right) f_{\mathbf{k}'}, \text{ for spin-triplets: } \Delta(\mathbf{k}) = (\mathbf{d}\cdot\mathbf{s}) is^y f_{\mathbf{k}} \end{cases} \tag{84}$$

Since $V^{\rm S}_{\mathbf{k},\mathbf{k}'}$ is always positive by definition, this means that for spin-singlets, a non-nodal s-wave solution (where $f_{\mathbf{k}}$ is a constant independent of $\mathbf{k}$) is allowed when only CDW fluctuations are present. One might expect that this solution corresponds to the highest T_c for a given $\xi_{\rm IVC}$, as the order-parameter is not required to modulate in real-space - this is also seen in our numerical results. For spin-triplets we can again focus on the limit near $\mathbf{k}+\mathbf{k}'=0$, since this is where $V^{\rm S}_{\mathbf{k},\mathbf{k}'}$ is maximized. Since $V^{\rm S}$ is repulsive in the triplet Cooper channel, this means that the gap equation can be satisfied when the gap-function changes sign, i.e, $f_{\mathbf{k}'=-\mathbf{k}} = -f_{\mathbf{k}}$, indicating unconventional pairing. However, these channels will typically have lower T_c than the spin-singlet s-wave channel discussed above, as the order parameter modulates in real space.

For the triplet IVC channel, the BCS decoupling takes the following form:

$$-\frac{1}{2A}\sum_{\mathbf{q}} g^{\rm T}_{\mathbf{q}} \,{\rm Tr}\left[n^{\rm IV}_{\rm T}(\mathbf{q})[n^{\rm IV}_{\rm T}(\mathbf{q})]^\dagger\right] \xrightarrow{\text{mean field}} \sum_{\mathbf{k},s,s'} \left[(s^i)^T\Delta^*(\mathbf{k})s^i\right]_{s's}\psi_{-,s,-\mathbf{k}'}\psi_{+,s',\mathbf{k}} + \left[s^i\Delta(\mathbf{k})(s^i)^T\right]_{ss'}\psi^\dagger_{+,s,\mathbf{k}}\psi^\dagger_{-,s',-\mathbf{k}'}$$
$$-A\sum_{\mathbf{k},\mathbf{k}'}[V^{\rm T}_{\mathbf{k},\mathbf{k}'}]^{-1}\,{\rm Tr}\left[(s^i)^T\Delta^*(\mathbf{k})s^i\Delta(\mathbf{k}')\right]$$
$$\text{where } V^{\rm T}_{\mathbf{k},\mathbf{k}'} = \frac{1}{2}g^{\rm T}_{\mathbf{q}=-\mathbf{k}-\mathbf{k}'}|\lambda^{+-}_{\mathbf{q}=-(\mathbf{k}+\mathbf{k}')}(\mathbf{k})|^2 \text{ and } \Delta_{ss'}(\mathbf{k}) = \frac{1}{A}\sum_{\mathbf{k}} V^{\rm T}_{\mathbf{k},\mathbf{k}'}\langle\psi_{-,s,-\mathbf{k}'}\psi_{+,s',\mathbf{k}}\rangle \tag{85}$$

Therefore, the total mean-field Hamiltonian can be written as:

$$H_{MF} = H_{\rm BdG} - A\sum_{\mathbf{k},\mathbf{k}'}[V^{\rm T}_{\mathbf{k},\mathbf{k}'}]^{-1}\,{\rm Tr}\left[(s^i)^T\Delta^*(\mathbf{k})s^i\Delta(\mathbf{k}')\right], \text{ where}$$
$$H_{\rm BdG} = \sum_{\mathbf{k},s,s'} \begin{pmatrix}\psi^\dagger_{+,s,\mathbf{k}} & \psi_{-,s',-\mathbf{k}}\end{pmatrix} \begin{pmatrix}\xi_{+,\mathbf{k}} & \left[s^i\Delta(\mathbf{k})(s^i)^T\right]_{ss'} \\ \left[(s^i)^T\Delta^*(\mathbf{k})s^i\right]_{ss'} & -\xi_-(-\mathbf{k})\end{pmatrix} \begin{pmatrix}\psi_{+,s,\mathbf{k}} \\ \psi^\dagger_{-,s',-\mathbf{k}}\end{pmatrix} \tag{86}$$

and summation on $i = x, y, z$ is implied. Evaluating the free energy and minimizing it leads to:

$$\Delta_{s's}(\mathbf{k}) = -\frac{1}{A}\sum_{\mathbf{k}'} V^{\rm T}_{\mathbf{k},\mathbf{k}'}\left(\frac{\tanh(\beta\xi_+(\mathbf{k}')/2)}{2\xi_+(\mathbf{k}')}\right)\left[s^i\Delta^{\rm T}(\mathbf{k}')(s^i)^T\right]_{ss'} \tag{87}$$

In turn, this implies the following equation for the spatial wave-functions of spin-singlet and spin-triplet superconductors:

$$f_{\mathbf{k}} = \begin{cases} -\frac{3}{A}\sum_{\mathbf{k}'} V^{\rm T}_{\mathbf{k},\mathbf{k}'}\left(\frac{\tanh\left(\beta\xi_+(\mathbf{k}')/2\right)}{2\xi_+(\mathbf{k}')}\right) f_{\mathbf{k}'}, \text{ for spin-singlets: } \Delta(\mathbf{k}) = is^y f_{\mathbf{k}} \\ -\frac{1}{A}\sum_{\mathbf{k}'} V^{\rm T}_{\mathbf{k},\mathbf{k}'}\left(\frac{\tanh\left(\beta\xi_+(\mathbf{k}')/2\right)}{2\xi_+(\mathbf{k}')}\right) f_{\mathbf{k}'}, \text{ for spin-triplets: } \Delta(\mathbf{k}) = (\mathbf{d}\cdot\mathbf{s}) is^y f_{\mathbf{k}} \end{cases} \tag{88}$$

We see that the interaction $V^{\rm T}$ is repulsive both in the spin-singlet and spin-triplet channels, hence a non-nodal s-wave superconductor is not favored. However, pair-correlation with $f_{\mathbf{k}'=-\mathbf{k}} = -f_{\mathbf{k}}$, i.e, unconventional superconductivity

will be favored in both cases, but because of the additional factor of 3 coming from the spin-trace, T_c will be larger for the spin-singlets. Hence, the preferred channel due to SDW fluctuations is either a gapped chiral, or a nodal non-chiral, spin-singlet superconductor.

Combining the results from the analysis of CDW and SDW fluctuations, we find that when both are present, $f_{\mathbf{k}}$ is given by the self-consistent solution of the following equations.

$$f_{\mathbf{k}} = \begin{cases} -\frac{1}{2A}\sum_{\mathbf{k}'}(3g^{\mathrm{T}}_{\mathbf{q}=-\mathbf{k}-\mathbf{k}'} - g^{\mathrm{S}}_{\mathbf{q}=-\mathbf{k}-\mathbf{k}'})|\lambda^{+-}_{\mathbf{q}=-\mathbf{k}-\mathbf{k}'}(\mathbf{k})|^2 \left(\frac{\tanh\left(\beta\xi_+(\mathbf{k}')/2\right)}{2\xi_+(\mathbf{k}')}\right) f_{\mathbf{k}'}, \text{ for spin-singlets: } \Delta(\mathbf{k}) = is^y f_{\mathbf{k}} \\ -\frac{1}{2A}\sum_{\mathbf{k}'}(g^{\mathrm{T}}_{\mathbf{q}=-\mathbf{k}-\mathbf{k}'} + g^{\mathrm{S}}_{\mathbf{q}=-\mathbf{k}-\mathbf{k}'})|\lambda^{+-}_{\mathbf{q}=-\mathbf{k}-\mathbf{k}'}(\mathbf{k})|^2 \left(\frac{\tanh\left(\beta\xi_+(\mathbf{k}')/2\right)}{2\xi_+(\mathbf{k}')}\right) f_{\mathbf{k}'}, \text{ for spin-triplets: } \Delta(\mathbf{k}) = (\mathbf{d}\cdot\mathbf{s}) is^y f_{\mathbf{k}} \end{cases} \tag{89}$$

Eq. (89) constitute the generalizations of the mean-field analysis in Eq. (11) in the main text, and identical conclusions regarding the preferred superconducting channels are reached from both sets of equations.

To end this section, we carry out an identical analysis for the gate-screened $\mathrm{SU}(2)_+\times$ $\mathrm{SU}(2)_-$-symmetric Coulomb repulsion $V_C(\mathbf{q})$, which we recall below.

$$H_C = \frac{1}{2A}\sum V_C(|\mathbf{q}|) : \rho(\mathbf{q})\ \rho(-\mathbf{q}) : \tag{90}$$

The BCS decoupling takes the following form:

$$H_C \xrightarrow{\mathbf{q}=\mathbf{k}-\mathbf{k}'} \frac{1}{A}\sum_{\mathbf{k},\mathbf{k}'} V^c_{\mathbf{k},\mathbf{k}'} \psi^\dagger_{+,s,\mathbf{k}} \psi^\dagger_{-,s',-\mathbf{k}} \psi_{-,s',-\mathbf{k}'} \psi_{+,s,\mathbf{k}'}, \text{ with } V^c_{\mathbf{k},\mathbf{k}'} = |\lambda^{++}_{\mathbf{q}=\mathbf{k}'-\mathbf{k})}(\mathbf{k})|^2 V_C(|\mathbf{q}=\mathbf{k}'-\mathbf{k}|) \tag{91}$$

The structure of Eq. (91) is identical to that of Eq. (69), with $V_{\mathbf{k},\mathbf{k}'} \to V^c_{\mathbf{k},\mathbf{k}'}$. Therefore, the mean-field decoupling amd BdG Hamiltonian also follow Eqs. (72) and (73) respectively, with the same replacement. In turn, the gap equation leads to the following condition for the spatial wave-functions for both spin-singlet and spin-triplet superconductors, which are degenerate due to the $\mathrm{SU}(2)_+\times$ $\mathrm{SU}(2)_-$ symmetry of the interaction.

$$f_{\mathbf{k}} = -\frac{1}{A}\sum_{\mathbf{k}'} V^c_{\mathbf{k},\mathbf{k}'} \left(\frac{\tanh(\beta\xi_+(\mathbf{k}')/2)}{2\xi_+(\mathbf{k}')}\right) f_{\mathbf{k}'} \tag{92}$$

Since $V^c_{\mathbf{k},\mathbf{k}'}$ is maximized at $\mathbf{k} = \mathbf{k}'$ (corresponding to $\mathbf{q} = 0$), we focus on this limit and note that generally such a gap equation does not have a solution, at least for a single Fermi surface. However, in presence of multiple Fermi surfaces (eg: corresponding to an annular Fermi sea) and/or in presence of other mechanisms for attractive pairing (eg: IVC fluctuations), there may exist a solution which have opposite signs on the inner and outer rings. In practice, we find that this is indeed the case when both IVC fluctuations and RPA-screened Coulomb repulsion ($V_C \to V_{\mathrm{RPA}}$, see Eq. 8) are considered in tandem, as was done for the plots in Fig. 3(a,e) in the main text.

C. Estimate of the coupling strength g

In this section, we provide a rough estimate the phenomenological coupling strength g which appeared in the effective IVC Hamiltonian in Eq. (6) in the main text. For this purpose, we start with the long-range Coulomb interaction H_C and consider random phase approximation (RPA) corrections to it by itinerant fermions, as described in Eq. (8). We now decouple it into the IVC channel, since strong IVC fluctuations constitute the mechanism of superconductivity in our picture:

$$H_C = \frac{1}{2A}\sum_{\mathbf{q}} V_{\mathrm{RPA}}(\mathbf{q}) : \rho(\mathbf{q})\rho(-\mathbf{q}) : \xrightarrow{\text{decoupled to IVC channel}} = -\frac{1}{2A}\sum_{\mathbf{q}} V_{\mathrm{RPA}}(\mathbf{q})\,\mathrm{Tr}\left[n^{\mathrm{IV}}(\mathbf{q})[n^{\mathrm{IV}}(\mathbf{q})]^\dagger\right] + \cdots \tag{93}$$

and the $\cdots$ denote terms which involve Coulomb repulsion between electrons in the same valley, and are expected to be unimportant close to a phase transition to an IVC phase. Now we assume that the bare interaction is sufficiently gate-screened so that we can approximate $V_C(\mathbf{q}) \approx V_0$, and consequently the RPA screened interaction takes the following form (setting the O(1) constant $c = 1$):

$$V_{\mathrm{RPA}}(\mathbf{q}) = \frac{V_0}{1 + V_0\chi_{\rho\rho}(\mathbf{q})} \approx \frac{V_0}{1 - V_0\,\chi_0(1 - \mathbf{q}^2/k_F^2)} = \frac{k_F^2/\chi_0}{\frac{(1-V_0\chi_0)k_F^2}{V_0\chi_0} + \mathbf{q}^2} \equiv \frac{k_F^2/\chi_0}{\mathbf{q}^2 + \xi_{\mathrm{IVC}}^{-2}} \tag{94}$$

where $\chi_0 = -\chi_{\rho\rho}(\mathbf{0}) \approx \partial_\mu n_e$ is approximately the density of states at the Fermi surface(s), and $\xi_{\mathrm{IVC}}^{-2} \equiv [(V_0\chi_0)^{-1} - 1]k_F^2$ is the squared inverse correlation length set by the distance from the IVC instability that sets in at $V_0\chi_0 = 1$. Noting

the the Fermi momentum k_F is set by the density, i.e, $k_F \sim \sqrt{n_e}$, we conclude that when IVC fluctuations are large we can approximate:

$$H_C \approx H^{\text{eff}}_{\text{IVC}} + \cdots \,, \text{ where } H^{\text{eff}}_{\text{IVC}} = -\frac{1}{A}\sum_{\mathbf{q}} \frac{n_e/\chi_0}{\mathbf{q}^2 + \xi^{-2}_{\text{IVC}}} \operatorname{Tr}\left[n^{\text{IV}}(\mathbf{q})[n^{\text{IV}}(\mathbf{q})]^\dagger\right] \tag{95}$$

whereby we identify $g = n_e/\chi_0$.

As mentioned in the main text, our analysis does not take into account the frequency dependence of the effective interaction (94) and the damping of the electrons by IVC fluctuations. These effects become important sufficiently close to the critical point [17] (i.e., for sufficiently large $k_F\xi_{\text{IVC}}$), and our treatment is expected to break down. We leave a full treatment of pairing in the quantum critical regime to future work.

D. Estimate of the IVC correlation length ξ_{IVC}

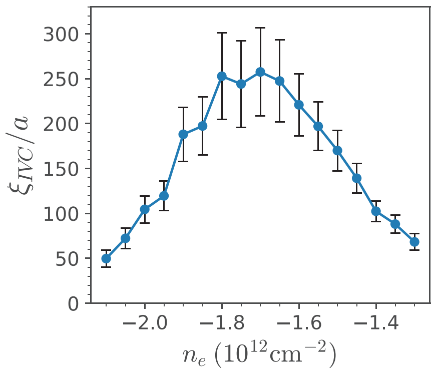

Supplementary Figure 7. IVC correlation length ξ_{IVC} obtained from self-consistent HF wavefunction using $\chi_0 = 0.16\,\text{eV}^{-1}$ per unit cell, at $u = 30$ meV and $\epsilon = 4.4$. The error bars indicate the least square error obtained from the fitting procedure. ξ_{IVC}/a remains around 100 for a significant range of carrier density n_e and peaks around $n_e = -1.7 \times 10^{12}$ cm^{-2}.

In this section, we numerically calculate the IVC correlation length ξ_{IVC} using the Slater determinant wavefunction obtained in self-consistent Hartree-Fock calculations. We first prepare a fully symmetric Slater determinant ground state $|\phi_{sym}\rangle$ using self-consistent HF on a 151×151 momentum grid with $u = 30$ meV, $\epsilon = 4.4$, and $\chi_0 = 0.16\,\text{eV}^{-1}$ per unit cell. There exists a gapped pseudo-Goldstone mode $|\phi^x_{\mathbf{q}}\rangle$ that corresponds to the breaking of valley $\text{U}(1)_v$ symmetry,

$$|\phi^x_{\mathbf{q}}\rangle = \left(\sum_{\mathbf{k}} c^\dagger_{+,\mathbf{k}} c_{-,\mathbf{k}+\mathbf{q}} + \text{h.c.}\right)|\phi_{sym}\rangle \tag{96}$$

We compute the dispersion of this mode above the ground state energy using the single-mode approximation:

$$E_{\mathbf{q}} = \frac{\langle \phi^x_{\mathbf{q}}|H|\phi^x_{\mathbf{q}}\rangle}{\langle \phi^x_{\mathbf{q}}|\phi^x_{\mathbf{q}}\rangle} - \langle\phi_{sym}|H|\phi_{sym}\rangle \tag{97}$$

where H is the full interacting Hamiltonian. At small momentum, we expect the dispersion to take the form of $E_{\mathbf{q}} = v\sqrt{\mathbf{q}^2 + \xi^{-2}_{\text{IVC}}}$, where v is the IVC mode velocity, and ξ^{-1}_{IVC} sets the 'mass gap' of IVC fluctuations. At a second order phase transition, ξ_{IVC} diverges as the fluctuations become truly gapless. However, they can also be nearly gapless for a weakly first order transition, indicated by a very large ξ_{IVC} (relative to lattice spacing a). In practice, we perform all calculations in terms of the projector $P_{\tau,\tau'}(\mathbf{k})$ (see Ref. 18 for details) and include the first order symmetry-allowed lattice harmonic in $E_{\mathbf{q}}$ for better fitting, i.e., we fit $E_{\mathbf{q}} = v\sqrt{\mathbf{q}^2 + \eta E_6(\mathbf{q}) + \xi^{-2}_{IVC}}$, where the lattice harmonic $E_6(\mathbf{q}) = \text{Re}[(q_x + iq_y)^6]/(q_x^2 + q_y^2)^3 + 1$ is a bounded positive function of q independent of its magnitude. Typically, we find $v \approx 10^4$ m/s and $\eta \sim 10^{-5}/a^2$, indicating that lattice effects are quite small. As a sanity check, we have also confirmed that the actual Goldstone mode $|\phi^z_{\mathbf{q}}\rangle = \sum_{\tau,\mathbf{k}}(-1)^\tau c^\dagger_{\tau,\mathbf{k}} c_{\tau,\mathbf{k}+\mathbf{q}}\,|\phi_{IVC}\rangle$ is always gapless with linear dispersion once we are within the IVC phase.

Our results are summarized in Supplementary Figure 7. While the single mode approximation is not expected to be particularly accurate very close to the transition, we nevertheless find that the IVC correlation length ξ_{IVC} remains around $100a$ in a significant range of hole-density. Based on our numerical solution to the gap equation, the superconducting transition temperature T_c will be around $0.1 - 1\,\mathrm{K}$ in such filling range (see Fig. 3(e) in the main text), consistent with experimental observations.

E. Impact of Hund's coupling on superconductivity

The Hund's coupling term in Eq. (45) amplifies certain IVC fluctuations, and thus can aid certain pairing channel. In this section, we show that a spatially local Hund's coupling does not affect p/f-wave superconductors, but non-local Hund's coupling can indeed aid certain pairing channels in the same manner as IVC fluctuations. To do so, we start by decoupling the Hund's term in the superconducting channel, to see which channel it aids among the ones induced by IVC fluctuations.

$$\langle H_{\text{Hund's}} \rangle = \frac{1}{A} \sum_{\mathbf{k},\mathbf{k}'} J_H(\mathbf{q} = -\mathbf{k} - \mathbf{k}') |\lambda^{+-}_{\mathbf{q}=-\mathbf{k}-\mathbf{k}'}(\mathbf{k})|^2 \,\mathrm{Tr}\big[F^*(\mathbf{k}) s^i F(\mathbf{k}')(s^i)^T\big] \tag{98}$$

If the Hund's coupling was local in real space, and the projected form-factor $\lambda^{+-}_{\mathbf{q}}(\mathbf{k})$ was featureles (eg: as in the sublattice-polarized case), these would be approximately independent of $\mathbf{q}$. In such a scenario, the Hund's coupling cannot affect the degeneracy between spin-singlet and triplet, as the orbital wavefunction (in the preferred channels from IVC fluctuations) vanishes when the two electrons in the Cooper pair approach the same point in space. This can be explicitly seen by setting $F(\mathbf{k}) = is^y f_{\mathbf{k}}$ (spin-singlet) or $F_{\mathbf{k}} = is^y(\mathbf{d}\cdot\mathbf{s})f_{\mathbf{k}}$ (spin-triplet) in Eq. (98) implies that $H_{\text{Hund's}} \propto J_H |\sum_{\mathbf{k}} f_{\mathbf{k}}|^2$, which evaluates to zero as long as $f_{-\mathbf{k}} = -f_{\mathbf{k}}$.

However, in reality the Hund's term need not be so local, and the valence-band wave-functions of the electrons are also extended in real space. So we may consider the opposite limit of $\mathbf{q} = 0$ in $J_H(\mathbf{q})$:

$$\mathrm{Tr}\big[F^\dagger(\mathbf{k}) s^i F(\mathbf{k})(s^i)^T\big] = \begin{cases} 6 f^*_{\mathbf{k}} f_{-\mathbf{k}}, & \text{if } F_{\mathbf{k}} = is^y f_{\mathbf{k}} \\ 2 f^*_{\mathbf{k}} f_{-\mathbf{k}}, & \text{if } F_{\mathbf{k}} = is^y(\mathbf{d}\cdot\mathbf{s}) f_{\mathbf{k}} \end{cases} \tag{99}$$

So for ferromagnetic Hund's ($J_H > 0$) which prefers the SDW IVC, chosen states are spin-singlets. This is not surprising, as $H_{\text{Hund's}}$ and H_{IVC} are basically identical near $\mathbf{q} = \mathbf{0}$ upto constant pre-factors. For AF Hund's ($J_H < 0$) which prefers CDW IVC, will choose a non-nodal spin-singlet s-wave state just by itself. However, when IVC fluctuations are near-critical, AF Hund's will instead perturbatively prefer a spin-triplet state by amplifying CDW fluctuations, as discussed in the main text.

Next, we consider the spin-polarized case, we have (with $\mathbf{d} = (1, i, 0)/\sqrt{2}$):

$$\langle H_{\text{Hund's}} \rangle = \frac{1}{A} \sum_{\mathbf{k},\mathbf{k}'} J_H(\mathbf{q} = -\mathbf{k} - \mathbf{k}') |\lambda^{+-}_{\mathbf{q}=-\mathbf{k}-\mathbf{k}'}(\mathbf{k})|^2 f^*_{\mathbf{k}} f_{\mathbf{k}'} \tag{100}$$

Analyzing the $\mathbf{q} = \mathbf{0}$ scenario again prefers a gapped chiral or a nodal non-chiral superconductor.

Finally, we comment on the other symmetry allowed Hund's coupling term $\tilde{J}_H$ (as in Eq. (46)), although we believe its magnitude is quite small. This kind of Hund's term has been considered in the context of superconductivity in twisted bilayer graphene [19, 20]. Such a term can also be decomposed into the pairing channel, but its effects are quite different from J_H.

$$\langle \tilde{H}_{\text{Hund's}} \rangle = -\frac{1}{A} \sum_{\mathbf{k},\mathbf{k}'} \tilde{J}_H(\mathbf{q} = \mathbf{k}' - \mathbf{k}) |\lambda^{++}_{\mathbf{q}=\mathbf{k}'-\mathbf{k}}(\mathbf{k})|^2 \,\mathrm{Tr}\big[F^\dagger(\mathbf{k}) s^i F(\mathbf{k}')(s^i)^T\big] \tag{101}$$

Once again, a local Hund's term cannot affect a pairing wave-function which is spatially antisymmetric. However, it can affect a pairing wave-function that is spatially symmetric. Considering the $\mathbf{q} \to 0$ limit as before, we see:

$$\mathrm{Tr}\big[F^\dagger(\mathbf{k}) s^i F(\mathbf{k})(s^i)^T\big] = \begin{cases} -6 f^*_{\mathbf{k}} f_{\mathbf{k}}, & \text{if } F_{\mathbf{k}} = is^y f_{\mathbf{k}} \\ 2 f^*_{\mathbf{k}} f_{\mathbf{k}}, & \text{if } F_{\mathbf{k}} = is^y(\mathbf{d}\cdot\mathbf{s}) f_{\mathbf{k}} \end{cases} \tag{102}$$

Thus, such a Hund's term will always prefer $f_{-\mathbf{k}} = f_{\mathbf{k}}$, i.e, s-wave pairing (as it has the least number of nodes). For ferromagnetic Hund's ($\tilde{J}_H > 0$, weakly prefers the triplet or SDW IVC), $\tilde{H}_{\text{Hund's}}$ prefers spin-triplet superconductivity. For AF Hund's ($\tilde{J}_H < 0$, weakly prefers the singlet or CDW IVC), $\tilde{H}_{\text{Hund's}}$ prefers spin-singlet superconductivity.

Finally, FM Hund's $\tilde{J}_H > 0$ can also spin-polarize the system. In this case, pairing will be mediated between the same spin species (assumed up-spin) with $F(\mathbf{k}) = f_{\mathbf{k}}\delta_{s,\uparrow}\delta_{s',\uparrow}$ we find that:

$$\mathrm{Tr}\left[F^\dagger(\mathbf{k})s^i F(\mathbf{k})(s^i)^T\right] = 2f^*_{\mathbf{k}}f_{\mathbf{k}} \tag{103}$$

and the resultant superconductivity is a non-unitary s-wave spin-triplet that is aided by this kind of Hund's coupling if only $\tilde{J}_H > 0$ is present.

Thus, in conclusion, ferromagnetic (FM) Hund's ($J_H > 0$) which prefers the spin-triplet SDW IVC, will also choose chiral or nodal superconducting states, just like the IVC fluctuations, but prefer spin-singlets over triplets. AF Hund's ($J_H < 0$) which prefers spin-singlet CDW IVC, will perturbatively choose spin-triplet chiral or nodal states when it slightly amplifies CDW flucutations, with a crossover to a spin-singlet non-nodal s-wave states when it is the only term present when it largely magnifies SDW fluctuations. Finally the spin-polarized IVC is favored by FM Hund's, and thus Hund's would perturbatively favor the spin-polarized chiral or nodal superconductor that arises from spin-polarized IVC fluctuations. For the other kind of Hund's term with potentially small $\tilde{J}_H$, the major difference comes for the SDW IVC, where FM $\tilde{J}_H > 0$ picks a conventional s-wave spin-triplet, while FM $J_H > 0$ would pick an unconventional chiral or nodal spin-singlet.

[1] Fan Zhang, Bhagawan Sahu, Hongki Min, and A. H. MacDonald, "Band structure of *abc*-stacked graphene trilayers," Phys. Rev. B **82**, 035409 (2010).

[2] Haoxin Zhou, Tian Xie, Areg Ghazaryan, Tobias Holder, James R. Ehrets, Eric M. Spanton, Takashi Taniguchi, Kenji Watanabe, Erez Berg, Maksym Serbyn, and Andrea F. Young, "Half- and quarter-metals in rhombohedral trilayer graphene," Nature (London) **598**, 429–433 (2021), arXiv:2104.00653 [cond-mat.mes-hall].

[3] Sylvain Latil and Luc Henrard, "Charge carriers in few-layer graphene films," Phys. Rev. Lett. **97**, 036803 (2006).

[4] Vladimir Cvetkovic and Oskar Vafek, "Topology and symmetry breaking in abc trilayer graphene," (2012), arXiv:1210.4923 [cond-mat.str-el].

[5] Nick Bultinck, Eslam Khalaf, Shang Liu, Shubhayu Chatterjee, Ashvin Vishwanath, and Michael P. Zaletel, "Ground state and hidden symmetry of magic-angle graphene at even integer filling," Phys. Rev. X **10**, 031034 (2020).

[6] Konstantin N. Kudin, Gustavo E. Scuseria, and Eric Cancès, "A black-box self-consistent field convergence algorithm: One step closer," The Journal of Chemical Physics **116**, 8255–8261 (2002).

[7] Eric Cancès and Claude Le Bris, "Can we outperform the diis approach for electronic structure calculations?" International Journal of Quantum Chemistry **79**, 82–90.

[8] Piers Coleman, *Introduction to Many-Body Physics* (Cambridge University Press, 2015).

[9] Eslam Khalaf, Nick Bultinck, Ashvin Vishwanath, and Michael P. Zaletel, "Soft modes in magic angle twisted bilayer graphene," (2020), arXiv:2009.14827.

[10] Aaron L. Sharpe, Eli J. Fox, Arthur W. Barnard, Joe Finney, Kenji Watanabe, Takashi Taniguchi, M. A. Kastner, and David Goldhaber-Gordon, "Emergent ferromagnetism near three-quarters filling in twisted bilayer graphene," Science **365**, 605–608 (2019).

[11] M. Serlin, C. L. Tschirhart, H. Polshyn, Y. Zhang, J. Zhu, K. Watanabe, T. Taniguchi, L. Balents, and A. F. Young, "Intrinsic quantized anomalous hall effect in a moiré heterostructure," Science **367**, 900–903 (2019).

[12] Nick Bultinck, Shubhayu Chatterjee, and Michael P. Zaletel, "Mechanism for anomalous hall ferromagnetism in twisted bilayer graphene," Physical Review Letters **124** (2020), 10.1103/physrevlett.124.166601.

[13] Ya-Hui Zhang, Dan Mao, and T. Senthil, "Twisted bilayer graphene aligned with hexagonal boron nitride: Anomalous hall effect and a lattice model," Physical Review Research **1** (2019), 10.1103/physrevresearch.1.033126.

[14] I. L. Aleiner, D. E. Kharzeev, and A. M. Tsvelik, "Spontaneous symmetry breaking in graphene subjected to an in-plane magnetic field," Physical Review B **76** (2007).

[15] Haoxin Zhou, Tian Xie, Takashi Taniguchi, Kenji Watanabe, and Andrea F. Young, "Superconductivity in rhombohedral trilayer graphene," Nature (London) **598**, 434–438 (2021), arXiv:2106.07640 [cond-mat.mes-hall].

[16] Maxim Kharitonov, "Phase diagram for the $\nu = 0$ quantum hall state in monolayer graphene," Physical Review B **85** (2012), 10.1103/physrevb.85.155439.

[17] Ar Abanov, Andrey V Chubukov, and AM Finkel'stein, "Coherent vs. incoherent pairing in 2d systems near magnetic instability," EPL (Europhysics Letters) **54**, 488 (2001).

[18] Eslam Khalaf, Nick Bultinck, Ashvin Vishwanath, and Michael P. Zaletel, "Soft modes in magic angle twisted bilayer graphene," (2020), arXiv:2009.14827.

[19] Yi-Zhuang You and Ashvin Vishwanath, "Superconductivity from valley fluctuations and approximate SO(4) symmetry in a weak coupling theory of twisted bilayer graphene," npj Quantum Materials **4**, 16 (2019), arXiv:1805.06867 [cond-mat.str-el].

[20] J. F. Dodaro, S. A. Kivelson, Y. Schattner, X. Q. Sun, and C. Wang, "Phases of a phenomenological model of twisted bilayer graphene," Phys. Rev. B **98**, 075154 (2018).
